# Association between low lung function and the increased risk of age-related macular degeneration: A population-based prospective cohort study

**DOI:** 10.7189/jogh.14.04102

**Published:** 2024-06-07

**Authors:** Guanran Zhang, Yanlin Qu, Zhenyu Wu, Wenjia Liu, Huihuan Luo, Renjie Chen, Huixun Jia, Xiaodong Sun

**Affiliations:** 1 Department of Ophthalmology, Shanghai General Hospital, Shanghai Jiao Tong University School of Medicine, Shanghai, China; 2 Shanghai Key Laboratory of Ocular Fundus Diseases, Shanghai, China; 3 National Clinical Research Center for Eye Diseases, Shanghai, China; 4 School of Public Health, Fudan University, Shanghai, China; 5 Department of Biostatistics, School of Public Health, Key Laboratory of Public Health Safety and Collaborative Innovation Center of Social Risks Governance in Health, Fudan University, Shanghai, China

## Abstract

**Background:**

Low lung function is associated with an increased risk of age-related diseases. However, the relationship between age-related macular degeneration (AMD), the leading cause of blindness, and lung function remains unclear. We aimed to investigate whether low lung function increases the risk of AMD and the potential mechanisms behind this association.

**Methods:**

We conducted a prospective cohort analysis of 409 230 UK Biobank participants with completed lung function after excluding individuals with AMD. We used Cox proportional hazards models to estimate the risk of AMD incidence and mediation models to explore potential mechanisms driven by inflammatory markers, erythrocyte-related measures, and metabolites.

**Results:**

Overall, 6477 AMD cases were diagnosed across an average of 12.4 years of follow-up. Participants with low lung function had an increased risk of developing AMD compared to those with high lung function (forced vital capacity: adjusted hazard ratio (aHR) = 1.20 (95% confidence interval (CI) = 1.07–1.34); forced expiratory volume in one second: aHR = 1.32 (95% CI = 1.18–1.47); peak expiratory flow: aHR = 1.32 (95% CI = 1.20–1.45)). Inflammatory markers and erythrocyte-related measures mediated this relationship, acting as a pathway through which low lung function influenced AMD. The interactions of body mass index (BMI), sex, and smoking were significant and the effect of lung function on AMD was higher in men, obese, and smoking populations.

**Conclusions:**

The increased risk of AMD was associated with low lung function, with inflammatory and erythrocyte-related markers mediating this relationship. This suggests that improvements in lung function could reduce the risk of AMD, thereby promoting health and longevity.

Age-related macular degeneration (AMD) is the main cause of blindness in developed countries, affecting approximately 200 million individuals globally and presenting an important health concern [1]. With this widespread prevalence, the disease contributes significantly to the global disease burden and to widening health disparities [2]. Beyond physical health, AMD also affects social well-being through its negative impact on quality of life, functional abilities, and depression rates [3]. Due to its irreversible nature and the absence of effective prevention strategies, investigating the modifiable risk factors and understanding the mechanisms leading to AMD is essential for promoting health and longevity [4,5].

Alongside smoking, obesity, physical activity, dietary behaviours, high blood pressure, and lipid levels, hypoxia has been associated with the development and progression of AMD [4,6–8]. Relatedly, due to its direct link to hypoxaemia function, low lung function has also emerged recently as a potential modifiable risk factor for AMD [9-12]. However, previous studies that suggested low lung function may pose a risk for AMD were limited by their cross-sectional design [13–15] or small sample sizes and incomplete adjustments for confounding factors [16–18]. This leaves a need for large-sample cohort studies with adequate covariates that would comprehensively investigate this proposed link. Moreover, most existing studies were based on association analyses, meaning they did not explore the mechanisms linking lung function, biomarkers, and AMD which could inform the treatment and prevention of AMD.

To address these research gaps, we performed an association analysis to examine whether low lung function contributed to an increased risk of AMD using population-based data from the UK Biobank, after which we investigated the underlying pathways using biomarker data in order to explore the mechanisms through which lung function may precipitate AMD.

## METHODS

### Study population

Designed as a population-based prospective cohort study, the UK Biobank recruited over 500 000 participants aged 37–73 years from 22 assessment centres in England, Wales, and Scotland between 2006 and 2010 [19,20] (**Online Supplementary Document**). In our study, we excluded those with missing information on lung function measures (n = 48 800), those with missing data of detections of laboratory blood tests (n = 43 979), and those with AMD at baseline (n = 367), leaving 409 230 individuals for further analyses.

The National Information Governance Board for Health and Social Care and the National Health Service North West Multicenter Research Ethics Committee approved the protocols of the initial UK Biobank study. All participants provided informed written consent on their first visit to the assessment centres. Therefore, we sought no ethical approval for our study.

### Lung function measures

In the absence of contraindications, all individuals were required to accomplish pre-bronchodilator spirometry at recruitment. Two or three blows of forced vital capacity (FVC), forced expiratory volume in 1 second (FEV1), and peak expiratory flow (PEF) were recorded within six minutes using a spirometer (Pneumotrac 6800; Vitalograph, Buckingham, UK). The repeatability of the first two blows in FVC and FEV1 was evaluated, and an acceptable difference (≤5%) indicated that the third blow was not required [12]. The maximum acceptable blows were used [21]. Therefore, we examined the association of lung function (as evaluated by FVC, FEV1, and PEF) and the risk of AMD. Additionally, we divided participants into those with airflow obstruction; those with preserved ratio impaired spirometry (PRISm); and healthy controls, as proposed by previous research [12,22] (**Online Supplementary Document**).

### Outcome

Our primary outcome of interest was the diagnosis of AMD according to a combination of main and secondary International Classification of Diseases (ICD)-9 (3625) and ICD-10 (H35.3) codes, which covered all stages of AMD [23]. Participants were followed up from recruitment to initial AMD diagnosis, death, loss to follow-up, or the end of the study period (November 2021), whichever came first.

### Covariates

We retrieved the following data, collected by the UK Biobank study team collected data through questionnaires at baseline: sociodemographic factors (age, sex, race, education, employment status); body mass index (BMI); lifestyle factors (smoking, alcohol consumption, physical activity); Townsend deprivation index (TDI); self-reported diseases (hypertension, diabetes, stroke, angina, and heart attack); and environmental pollutants (particulate matter with aerodynamic diameters of <2.5 μm (PM_2.5_), and nitrogen dioxide (NO_2_)). The detailed definitions of alcohol consumption, physical activity, and environmental pollutants are described elsewhere [24,25].

### Inflammatory markers, erythrocyte-related measures, and plasma metabolites

To explore the potential mechanisms between lung function measures and the development of AMD, we retrieved data on inflammatory markers, erythrocyte-related measures, and plasma metabolites, which were collected through haematological assays at baseline [26,27]. Specifically, C reactive protein (CRP) and the counts or proportions of blood neutrophils, lymphocytes, monocytes, and platelets were collected as inflammatory markers. Following the methodology proposed by An et al. [28], we searched for neutrophil-to-lymphocyte ratio (NLR), lymphocytes-to-monocytes ratio (LMR), platelet-to-lymphocyte ratio (PLR), and systemic immune-inflammation index (SII) (neutrophils  ×  platelets/lymphocytes) to explore the systematic inflammatory status [11]. We also retrieved data on haematocrit percentage (HCT); haemoglobin concentration (Hb); erythrocyte and reticulocyte counts; and red blood cell distribution width (RDW). We also investigated blood oxygen-carrying capacity per the levels of erythrocyte-related measures. Overall, 168 metabolites, including fatty acids, lipoprotein lipids, and a series of low molecular weight metabolites, were measured for a subset of individuals (n = 98 625) using a high-throughput nuclear magnetic resonance (NMR)-based metabolic biomarker profiling platform.

### Statistical analyses

**Figure 1:**
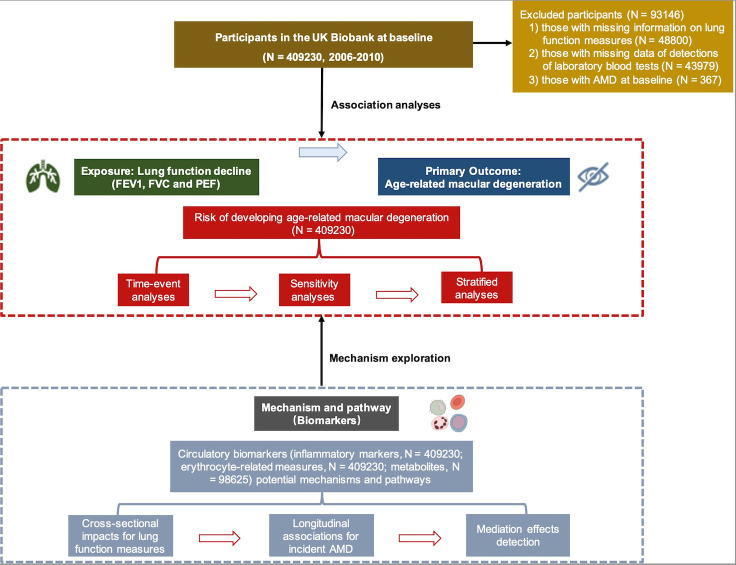
Study design and workflow. AMD – age-related macular degeneration, FEV1 – forced expiratory volume in 1 second, FVC – forced vital capacity, PEF – peak expiratory flow.

Our analysis had two parts: an examination of the impact of low lung function on incident AMD after performing a minus transformation for baseline lung function measurements (FVC, FEV1, and PEF) and an exploration of the biological mechanisms underlying these associations (**Figure 1**).

In the association analyses, we transformed lung function into quantiles, with the highest quantile (4th) set as the reference. In comparison, the first, second, and third quantiles could be considered as ‘low lung function.’ We then set up Cox proportional hazards models to estimate the risk of lung function on incident AMD and used restricted cubic splines (RCS) to explore whether the lung function-AMD associations were nonlinear. We established two models: model 1, adjusted for baseline age, sex, race, BMI, smoking and alcohol consumption status, physical activity, education level, and occupation status; and model 2 with additional adjustment for the height and disease histories. We reported our findings as adjusted hazard ratios (aHRs) and 95% confidence intervals (CIs).

Considering the effects of potential modifiers, we additional analyses stratified for sex, lifestyle factors (smoking, alcohol consumption, physical activity), and BMI. Moreover, we performed several sensitivity analyses to test the robustness of the results. To evaluate the confounding bias, we additionally regressed TDI, PM_2.5_, and NO_2_ in model 2, using available complete cases (n = 369 035). To control the selection bias, we re-analysed the associations between lung function measures and incident AMD by restricting the analyses to those with a baseline age of more than 50 years and to those of White ethnicity, because AMD was an age-related disease and individuals in the UK Biobank were mainly middle-aged Whites. To avoid reverse causation bias, we excluded those with AMD cases occurring in the first two years of follow-up. Finally, we performed the latent class analysis to identify any hidden variables driving the process that may not be measured easily.

We lastly explored biological markers in the mediation analyses. Specifically, we explored the impact of biomarkers for lung function levels and incident AMD via multivariate linear regression models or Cox proportional hazards models, respectively. We selected biomarkers with significant impacts on both lung functions and AMD to construct mediation models through the ‘lavaan’ package in R, version 4.1.3 (R Core Team, Vienna, Austria), adjusted for covariates in model 2.

We described baseline characteristics through means (x̄) with standard deviation (SDs); medians with interquartile ranges (IQRs); and frequencies with percentages, as appropriate. Alls hypothesis tests were two-sided, and we considered *P* < 0.05 as statistically significant. We adjusted *P*-values for false discovery rates in case of multiple testing. All analyses were performed using R, version 4.1.3 (R Core Team, Vienna, Austria).

## RESULTS

### Population characteristics

We included 409 230 participants in our study (**Table 1**). In total, 6477 AMD cases were diagnosed over 5 071 223 person-years of follow-up (x̄ = 12.7 years, maximum: 14.9 years). Most participants were older women; had lower educational levels, had higher levels of physical activity; and were smokers. Compared with those healthy controls, participants with incident AMD were more likely to have a history of diseases, including hypertension (*P* < 0.001), diabetes (*P* < 0.001), stroke (*P* < 0.001), angina (*P* < 0.001), and heart attack (*P* < 0.001). Patients with AMD had worsening lung functions compared with healthy controls.

**Table 1:** Baseline characteristics of participants by incident AMD*

	Without AMD (n = 402 754)	With AMD (n = 6477)	*P*-value
**Characteristics**			
Age in years, x̄ (SD)	56.3 (8.1)	62.9 (5.4)	<0.001
Men	183 806 (45.6)	2490 (38.4)	<0.001
White ethnicity or race	380 127 (94.8)	6144 (95.4)	0.038
Education			
*College or above*	152 851 (38.4)	2066 (32.3)	
*High school or equivalent*	179 400 (45.1)	2630 (41.2)	
*Less than high school*	65 933 (16.6)	1694 (26.5)	<0.001
Employed	369 075 (92.6)	6090 (95.0)	<0.001
BMI in kg/m^2^, x̄ (SD)	27.3 (4.7)	27.8 (4.8)	<0.001
Smoker	178 746 (44.6)	3221 (50.1)	<0.001
Alcohol consumption	131 258 (32.6)	2117 (32.7)	0.882
Regular physical activity	114 572 (28.5)	1734 (26.8)	0.003
Hypertension	93 994 (23.3)	2152 (33.2)	<0.001
Diabetes	19 488 (4.9)	608 (9.4)	<0.001
Stroke	5182 (1.3)	163 (2.5)	<0.001
Angina	10 355 (2.6)	332 (5.1)	<0.001
Heart attack	7643 (1.9)	194 (3.0)	<0.001
High PM_2.5_ exposure	172 621 (46.9)	2878 (46.8)	0.874
High NO_2_ exposure	49 835 (12.6)	804 (12.5)	0.972
Lung function measures			
*FVC in L*, x̄ (SD)	3.77 (1.09)	3.38 (1.11)	<0.001
*FEV1 in L*, x̄ (SD)	2.86 (0.80)	2.5 (0.72)	<0.001
*PEF in L/min*, x̄ (SD)	409.2 (127.3)	363.4 (118.1)	<0.001

AMD – age-related macular degeneration, BMI – body mass index, FEV1 – forced expiratory volume in 1 s, FVC – forced vital capacity, NO_2_ – nitrogen dioxide, PEF – peak expiratory flow, PM_2.5_ – particulate matter with aerodynamic diameters of <2.5 μm, SD – standard deviation, x̄ – mean

*Presented as n (%) unless specified otherwise.

### Associations of lung function on incident AMD

According to the RCS analyses, all lung function measures (FVC, FEV1, and PEF) had a linear effect on AMD (Figure S1 in the **Online Supplementary Document**). After adjusting for age and sex, race, BMI, smoking and alcohol consumption status, physical activity, education level, and employment status, individuals in the lower three quantiles had a significantly increased risk for incident AMD compared with those in the 4th quantile of FVC (first quantile: aHR = 1.23 (95% CI = 1.11–1.37); second quantile: aHR = 1.12 (95% CI = 1.01–1.23), third quantile: aHR = 1.14 (95% CI = 1.05–1.25)). We still observed these associations after additionally adjusting for height and the history of disease (first quantile: aHR = 1.12 (95% CI = 1.03–1.23); second quantile: aHR = 1.09 (95% CI = 0.99–1.21), third quantile: aHR = 1.20 (95% CI = 1.07–1.34)), as well as in the FEV1 and PEF analyses (**Table 2**).

**Table 2:** Association of lung function with AMD

Variables	AMD, n/N	Model 1, aHR (95% CI)*	Model 2, aHR (95% CI)†
FVC			
*FVC Q4*	898/102 044	ref	ref
*FVC Q3*	1357/101 843	1.14 (1.05–1.25)	1.12 (1.03–1.23)
*FVC Q2*	1611/101 943	1.12 (1.01–1.23)	1.09 (0.99–1.21)
*FVC Q1*	2611/103 400	1.23 (1.11–1.37)	1.20 (1.07–1.34)
*P for trend*	-	<0.001	0.004
FEV1			
*FEV1 Q4*	809/101 621	ref	ref
*FEV1 Q3*	1264/102 606	1.14 (1.04–1.25)	1.12 (1.02–1.23)
*FEV1 Q2*	1676/102 461	1.20 (1.09–1.32)	1.17 (1.06–1.30)
*FEV1 Q1*	2728/102 542	1.35 (1.21–1.49)	1.32 (1.18–1.47)
*P for trend*	-	<0.001	<0.001
PEF			
*PEF Q4*	946/101 916	ref	ref
*PEF Q3*	1290/101 349	1.13 (1.03–1.23)	1.11 (1.02–1.21)
*PEF Q2*	1706/103 199	1.22 (1.11–1.34)	1.20 (1.09–1.32)
*PEF Q1*	2535/102 766	1.35 (1.22–1.48)	1.32 (1.20–1.45)
*P value for trend*	-	<0.001	<0.001

aHR – adjusted hazard ratio, AMD – age-related macular degeneration, BMI – body mass index, CI – confidence interval, FEV1 – forced expiratory volume in 1 s, FVC – forced vital capacity, PEF – peak expiratory flow, Q – quantile

*Adjusted for baseline age and sex, race, BMI, smoking, alcohol consumption status, physical activity, education level, and occupation status.

†Adjusted for baseline age and sex, race, BMI, smoking, alcohol consumption status, physical activity, education level, occupation status, height history of hypertension, diabetes, stroke, angina, and heart attack.

We also defined PRISm and chronic obstructive pulmonary disease (COPD) based on the predicted FEV1 percentage and FEV1/FVC rate (Table S1 in the **Online Supplementary Document**). Compared with healthy controls, individuals with PRISm and COPD were both had a substantially increased risk for incident AMD at 12% (aHR = 1.12; 95% CI = 1.04–1.21, *P* = 0.004) and 23% (aHR = 1.23; 95% CI = 1.16–1.31, *P* < 0.001), respectively, after adjusting for variables in model 2. Our sensitivity analyses showed that lung function-AMD associations were robust in all scenarios (Tables S2–5 in the **Online Supplementary Document**).

### Analyses stratified for modifiers

**Figure 2:**
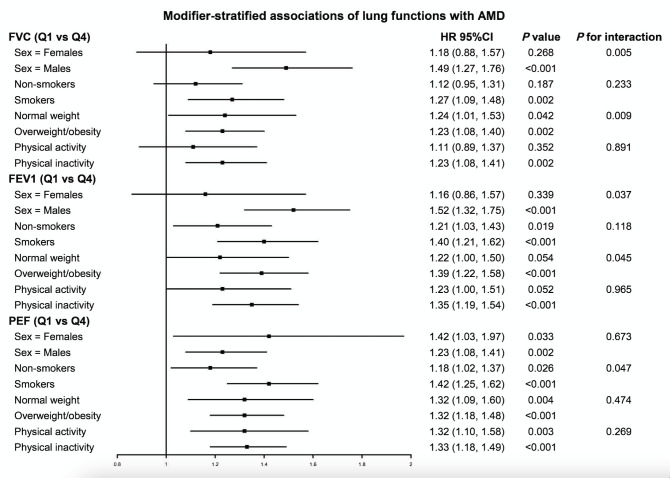
Associations between lung function and risk of incident AMD. Squares represent hazard ratios; horizontal lines indicate corresponding 95% CIs around aHRs. We calculated aHRs using Cox proportional hazards models after adjustments for baseline age and sex, race, BMI, smoking, alcohol consumption status, physical activity, education level, occupation status, height, history of hypertension, diabetes, stroke, angina, and heart attack. Only the aHRs (95% CIs) comparing those with the lowest lung function measures (Q1) against those with the highest (Q4) are shown. Overweight/obesity: BMI≥25 kg/m^2^; normal weight: BMI<25 kg/m^2^. AMD – age-related macular degeneration, CI – confidence interval, FEV1 – forced expiratory volume in 1 second, FVC – forced vital capacity, HR – hazard ratio, PEF – peak expiratory flow.

In view of potential modifiers, sex, smoking, physical activity, and BMI were significantly associated with AMD (Table S6 in the **Online Supplementary Document**), while alcohol consumption was not (*P* = 0.164 for model 1, *P* = 0.060 for model 2). Thus, we explored the interactions of the former, significantly associated variables with lung function in our stratified analyses (**Figure 2**). We only observed statistically significant interactions of both sex and BMI with FVC and FEV1, and of smoking status with PEF (*P*-value for interaction <0.05). Men with lower FVC and FEV1 levels had a higher risk of incident AMD than women. Likewise, compared with non-smokers (aaHR = 1.18; 955 CI = 1.02–1.37), smokers showed more serious adverse associations with AMD if they performed a worsening PEF (aaHR = 1.42; 95% CI = 1.25–1.62) (**Figure 2**).

The lung functions-AMD associations varied widely in different physical activity status subgroups, although the interaction effect was non-significant (**Figure 2**). Participants in the first quantile of FVC with regular physical activity were not associated with an increased risk for incident AMD (*P* = 0.352), while the effect in those who were physically inactive showed an aHR of 1.23 (95% CI = 1.08–1.41). We also observed these differences in patterns in our FEV1 and PEF analyses.

### Potential biological mechanisms for the associations between lung function and AMD

We observed significant associations of several inflammatory markers, erythrocyte-related measures, and blood metabolites with all three lung function levels, even after adjusting the *P*-values for false discovery rates. However, only a subset of them showed longitudinal associations with incident AMD, such as neutrophil count, lymphocyte percentage, CRP, SII, RDW, HCT, and Hb (Figures S2–4, Tables S7–11 in the **Online Supplementary Document**).

**Figure 3:**
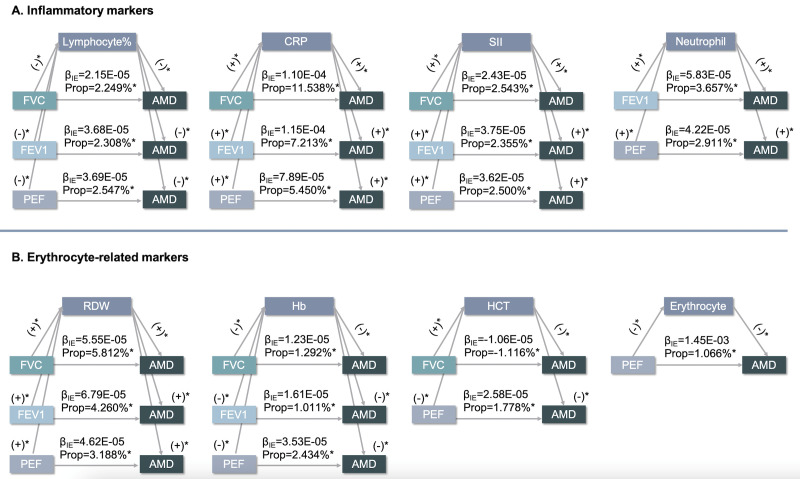
The mediation effects of peripheral immunity (**Panel A**) and erythrocyte-related (**Panel B**) measures on lung function-AMD associations. Plus symbol indicates a positive association. Minus symbol indicates a negative association. β_IE_ indicates the indirect effects of lung function measures on age-related macular degeneration. The lung function measures were minus-transformed. *P-value <0.05. CI – confidence interval, CRP – C reactive protein, FEV1 – forced expiratory volume in 1 second, FVC – forced vital capacity, Hb – hemoglobin concentration, HCT – hematocrit percentage, PEF – peak expiratory flow, RDW – red blood cell distribution width, SSI – systemic immune-inflammation index.

Lastly, we used mediation models to examine the potential biological mechanisms behind the lung function-AMD associations (**Figure 3**; Tables S12–14 in the **Online Supplementary Document**). After adjusting for variables in model 2, neutrophil count, lymphocyte percentage, C reactive protein, SII, erythrocyte count, RDW, HCT, and Hb emerged as significant mediators in the FVC-AMD relationship. All biomarkers partially elucidated the increased risk of AMD associated with reduced FVC (22.3%), FEV1 (23.2%), and PEF (25.0%).

## DISCUSSION

Based on data from the UK Biobank, we observed low lung function was associated with a significantly increased risk of AMD. This association was modified by sex, BMI, and smoking. Furthermore, the mediation analysis identified inflammatory and erythrocyte-related markers as pathways in the lung function-AMD relationship, improving our understanding of the lung function-AMD association. Based on our findings, the middle-aged and elderly populations should be screened for lung function as a preventive measure, while interventions should be designed to target obese men and smokers with compromised lung health to reduce their susceptibility to AMD.

Our findings align with those of prior studies, confirming that low lung function correlates with an increased risk of developing AMD later in life [13–18]. In the Beaver Dam Eye Study, women under 75 displayed a 79% increased risk of late-stage AMD in association with low PEF [15]. However, this study did not establish a clear temporal link between lung function and AMD. Chi-Yuan Li et al. [18] observed a higher risk of both exudative and non-exudative AMD within the COPD-afflicted cohort, with adjusted aHRs of 1.49 and 1.15, respectively. Nevertheless, their analyses did not account for potential confounders such as sex, race, smoking, and alcohol consumption, and was limited to the effects of COPD on AMD, overlooking those with relatively healthy lung function. In contrast, other studies have reported no connection between lung function and AMD [29,30]. Our prospective cohort study adjusted for these limitations, providing evidence that low lung function is a significant risk factor for AMD, thereby highlighting the clinical relevance of lung health in the prevention of ocular diseases. Furthermore, through the application of restricted cubic splines, we determined a linear negative relationship between lung function and AMD, suggesting that any intervention to improve lung health would help reduce the risk of AMD.

The progression of a chronic disease is an ongoing process, in which indicators of medical status, like hypoxia and inflammation, could potentially serve as mediators. Previous studies have explored the mediating pathways linking low lung function to higher risks of age-related diseases [11,31–35]. For example, a cohort study of over 400 000 individuals observed that low lung function could increase the risk of dementia [11]. Moreover, oral antioxidants were observed to significantly reduce the risk of cardiovascular diseases in patients with low lung function [34]. However, the mechanisms between lung function and AMD have yet to be fully clarified [2,4,5]. Potential explanations are that individuals with low lung function are more susceptible to oxygen insufficiency. Therefore the retina, given its highly oxygen-consuming nature, would probably be impacted. Here, hypoxia would trigger oxidative stress, subsequently activating downstream signals that ultimately lead to retinal damage, making it a key pathological factor in AMD [4]. In our study, four erythrocyte-related markers (RDW, Hb, HCT, and erythrocyte) mediated the mechanisms through which lung function increases the risk of AMD. Additionally, inflammation may also be involved in this progression, especially CRP. Nonetheless, this increased risk of AMD cannot be solely attributable to these biomarkers, underscoring the need to explore other mediating pathways between AMD and lung function. Specifically, further research could explore the potential link between plasma metabolites, lung function, and subsequent AMD.

We further identified interactions between lung function and factors including BMI, sex, and smoking, resulting in risk associations distinct from the overall pattern. Previous evidence has established that smoking increases the risk of AMD by two to four times [36,37]. Le Ma et al. [38] reported a 2% incremental increase in AMD risk for each additional 1 kg/m^2^ increase in BMI. Additionally, the inflammation and damage to alveolar epithelial cells induced by smoking could also impair lung function, which could also be exacerbated by obesity [39–42]. Hence, low lung function contributed most to AMD in men who were obese and who were smokers. This finding partially aligns with prior observations that the relationship between lung function and dementia is influenced by sex, BMI, and PM_2.5_ exposure [11].

Our study has several strengths. First, we included 409 230 participants, with an average follow-up duration of 12.4 years, providing extensive observations of participants experiencing incident AMD and ensuring sufficient statistical power for the subgroup analyses. Second, we effectively mitigated potential recall bias by using hospitalisation and mortality records to determine AMD status. We also retrieved a comprehensive set of variables, which ensured adequate covariate adjustments, subsequently minimising confounding bias. Importantly, we used quantitative mediation analysis to explore the mechanism linking low lung function with increased AMD risk. Further investigating this connection through biomarkers could further improve the design and implementation of public health strategies aimed at reducing AMD risk.

Nevertheless, this study also has several limitations. First, the UK Biobank cohort is predominantly Caucasian, a population which is both healthy and affluent, limiting the generalisability of our findings to other ethnic groups. Second, while our mediation analysis determined certain underlying mechanisms between lung function and AMD, it is constrained by the absence of cytokine data, a secondary but essential marker of inflammation, thereby hindering a better understanding of the broader underlying mechanisms. Third, although we rigorously adjusted for various covariates, we were unable to fully eliminate the effect of unmeasured confounding factors, particularly those related to genetic and familial characteristics. There is also a possibility that some of the included covariates were measured imprecisely. Lastly, we lacked data on serial lung function measurements and subtype-specific AMD, which currently warrant further studies.

## CONCLUSIONS

We observed a significant correlation between low lung function and an increased risk of AMD, which was modified by BMI, sex, and smoking, making obese men who are smokers a high-risk population. Moreover, blood biomarkers, reflecting hypoxia and inflammation, acted as mediators in and explained the potential mechanisms behind this relationship. Our study emphasises the critical role of optimal lung function in delaying or preventing the onset of AMD. Interventions aimed at improving lung health could hold significant potential for AMD prevention and have broader implications for overall health and longevity.

## Additional material


Online Supplementary Document
