# Supplementary material for: Association between low lung function and the increased risk of age-related macular degeneration: A population-based prospective cohort study
Source: J Glob Health. 2024 Jun 7;14:04102. doi: 10.7189/jogh.14.04102 (PMC11156252; doi:10.7189/jogh.14.04102)
Supplement: Online Supplementary Document [file jogh-14-04102-s001.pdf]

Association between low lung function and the increased risk of age-related macular degeneration: A population-based prospective cohort study

Supplementary material

Supplement methods

Table S1. Association between disease severity and risk of incident AMD.

Table S2. Sensitivity analyses for association between incident AMD and lung function.

Table S3. Association between risk for incident AMD and lung function in the White ethnicity.

Table S4. Average posterior probabilities, prevalence of latent classes, and item-response probabilities in four-latent class model

Table S5. Associations of the latent class of lung function with incident AMD

Table S6. Hazard ratios (95% CIs) of potential modifiers with incident AMD

Table S7. Blood biomarker characteristics in the UK Biobank study.

Table S8. Associations between lung function measures with inflammatory and erythrocyte-related markers at baseline.

Table S9. Associations between lung function measures with metabolites at baseline.

Table S10. Longitudinal associations between the risk of incident AMD with inflammatory and erythrocyte-related markers.

Table S11. Longitudinal associations between metabolites with the risk of incident AMD.

Table S12. The mediation effects of blood markers in the association between FVC and incident AMD risk.

Table S13. The mediation effects of blood markers in the association between FEV1 and incident AMD risk.

Table S14. The mediation effects of blood markers in the association between PEF and incident AMD risk.

Figure S1. Association of lung function as a continuous scale with incident AMD.

Figure S2. Heatmap of associations between blood inflammatory markers with lung function and incident AMD.

Figure S3. Heatmap of associations between erythrocyte-related measures with lung function and incident AMD.

Figure S4. Heatmap of associations between blood metabolites with lung function and incident AMD.

Supplement methods

Description of UK Biobank

The recruitment of participants including: (1) age between 40 and 69, (2) living within a reasonable travel distance from 22 assessment centers. Based on previous pilot experiences, it is estimated that around 5 million invitations will be sent to recruit 0.5 million participants. Email will be sourced from the National Health Service. Because our study is an observational study, randomization is not applicable [1].

Assessment of pulmonary diseases

We divided participants into airflow obstruction, preserved ratio impaired spirometry (PRISm), and healthy controls as proposed [2]. Specifically, airflow obstruction was defined as FEV1/FVC ratio < 0.70; PRISm was defined as a FEV1 of < 80% predicted and a FEV1/FVC ratio of ≥ 0.70 [2] according to the Global Initiative for Chronic Obstructive Lung Disease (COLD) criteria [3]; individuals with an FEV1 of ≥ 80% predicted and a FEV1/FVC rate of ≥ 0.7 were considered as healthy controls. The FEV1 percent predicted was estimated as per GLI-2012 values using the "RSpiro" package in R (version 4.1.3).

Air pollution estimates

Using a Land Use Regression (LUR) model, air pollutants including particulate matter (PM) with aerodynamic diameters of <2.5 μm ($PM_{2.5}$) and nitrogen dioxide (NO_2) were estimated for each address in UK Biobank. The average concentrations were calculated, annually. Information on NO_2 was collected between 2005-2007 and 2010, while the exposure data for $PM_{2.5}$ was measured in 2010. In the current study, the averaged mean levels of NO_2 were established to reflect the total exposure. Following previous study, participants were treated as those exposed to low or high pollution according to the World Health Organization yearly air quality guideline values or the median. ($PM_{2.5}$: low < 10 μg/m^3 or high ≥10 μg/m^3; NO_2: low < 40 μg/m^3 or high ≥40 μg/m^3) [4].

Assessment of alcohol drinking and Regular physical activity

Alcohol drinking: In the UK Biobank, participants were asked about the frequency of drinking alcohol, containing (almost) daily, three or four times a week, once or twice a week, one to three times a month, special occasions only, never, and prefer not to answer. Those who reported to drink alcohol would be asked about how much red wine (glasses), white wine (glasses), beer or cider

(pints), spirits or liqueurs (standard measures), fortified wine (glasses), and other alcoholic drinks (glasses) they consumed in an average month or week. We used the information to calculate the average units of alcohol each participant drank daily. A healthy level was defined as daily consumption of one drink or fewer for women and two drinks or fewer for men, according to the dietary guidelines in the UK (one drink contains 8 g in the UK) [5].

Regular physical activity: Frequency and time for physical activity (PA) were asked for individuals in the UK Biobank. In the current study, regular PA was defined to meet one of the following: (i) from the perspective of frequency, to engage in vigorous physical activity for at least one day and moderate activity for at least five days per week; (ii) from the perspective of time, to exercise of vigorous activity for at least 75 min or moderate activity for 150 min per week [6].

Hematology measurements

Four Beckman Coulter LH750 instruments were put to use in the analysis of samples collected in 4ml EDTA vacutainers from around participants comprising the UK Biobank baseline cohort (https://biobank.ndph.ox.ac.uk/ukb/ukb/docs/haematology.pdf). Every gathered sample underwent analysis through one or multiple LH750 instruments. Within 24 hours of the blood draw, these samples were scrutinized at the UK Biobank central laboratory. Before analyzing any participant samples, routine maintenance of the analyzers was conducted following the manufacturers' guidelines. Additionally, Quality Control (QC) assessments were executed on the systems. In the event that analyzers did not pass these QC checks, samples were withheld from measurement on that particular instrument until a thorough investigation was conducted and a successful resolution was implemented. Routine Preventative Maintenance (PM) procedures were conducted by Beckman, and any necessary service or repair tasks were also undertaken by Beckman as needed.

Metabolite measurements by NMR

EDTA plasma samples were assessed via Nightingale Health's nuclear magnetic resonance (NMR)-based metabolic biomarker profiling platform (https://biobank.ndph.ox.ac.uk/ukb/ukb/docs/nmrm_companion_doc.pdf). UK Biobank was responsible for the direct preparation of samples in 96-well plates. Employing TECAN Freedom EVO 150 robotic liquid handlers, a volume of no less than 85 μL of plasma was meticulously aliquoted into each well, boasting pipetting volume coefficients of variation below 0.75% across eight tips. Plasma samples, arranged in 96-well plates, were transported to Nightingale Health's laboratories in sample batches of approximately 5,000 to 20,000 on dry ice.

The meticulously prepared samples were placed onto a refrigerated sample changer, which ensures that samples in queue for measurement remain at a temperature of +6°C. Employing a 500 MHz NMR spectrometer (Bruker AVANCE IIIHD), two NMR spectra were captured for every individual plasma sample. The initial spectrum is a proton NMR spectrum pre-saturated, showcasing resonances primarily originating from proteins and lipids found within diverse lipoprotein particles. The second spectrum is a T2-relaxation-filtered rendition, designed to suppress the majority of the broad signals from macromolecules and lipoprotein lipids. This enhancement facilitates the detection of low-molecular-weight metabolites. Automated quality control was executed on the spectral data. The quantification of metabolic biomarkers was achieved through Nightingale Health's exclusive software (Nightingale Health biomarker quantification library 2020).

Nightingale Health's NMR platform for metabolic biomarker profiling ensures consistent results over time and across different spectrometers. Additionally, the metabolic biomarker platform by Nightingale Health has minimal sample preparation, eliminating the need for any extraction steps. These attributes lead to remarkably reproducible biomarker measurements. Pre-defined quality metrics were established through collaboration between UK Biobank and Nightingale Health to maintain uniform outcomes across the samples, and initial pilot measurements were undertaken. Nightingale Health conducted continuous real-time monitoring to ensure measurement consistency within and between spectrometers across the entire UK Biobank sample set. Each 96-well plate included two control samples supplied by Nightingale Health, serving as benchmarks for tracking consistency across multiple spectrometers. Moreover, in each well plate, two blind duplicate samples furnished by the UK Biobank were incorporated, their positional details revealed solely upon the delivery of results. Targets for the coefficient of variation (CV) were predetermined for the metabolic biomarker profile, encompassing both Nightingale Health's internal control samples and the blind duplicates from the UK Biobank. CVs for the majority of metabolic biomarkers remain below 5%. Nightingale Health's metabolic biomarker profiling platform is renowned for its consistent repeatability over time and its resistance to batch effects. As outlined in the guidance profiles offered by the UK Biobank, the metabolic biomarker data is generally suitable for epidemiological analyses without the need for pre-processing. These data can be examined using the

same methodologies as the clinical biochemistry data already accessible within the UK Biobank. (https://biobank.ndph.ox.ac.uk/ukb/ukb/docs/nmrm_companion_doc.pdf).

Reference

1. Sudlow C, Gallacher J, Allen N, et al. UK biobank: an open access resource for identifying the causes of a wide range of complex diseases of middle and old age. PLoS Med. 2015;12(3):e1001779. Published 2015 Mar 31. doi:10.1371/journal.pmed.1001779

2. Higbee DH, Granell R, Davey Smith G, Dodd JW. Prevalence, risk factors, and clinical implications of preserved ratio impaired spirometry: a UK Biobank cohort analysis. Lancet Respir Med. 2022 Feb;10(2):149-157.

3. Singh D, Agusti A, Anzueto A, Barnes PJ, Bourbeau J, Celli BR, Criner GJ, Frith P, Halpin DMG, Han M, López Varela MV, Martinez F, Montes de Oca M, Papi A, Pavord ID, Roche N, Sin DD, Stockley R, Vestbo J, Wedzicha JA, Vogelmeier C. Global Strategy for the Diagnosis, Management, and Prevention of Chronic Obstructive Lung Disease: the GOLD science committee report 2019. Eur Respir J. 2019 May 18;53(5):1900164.

4. WHO global air quality guidelines: Particulate matter (PM2.5 and PM10), ozone, nitrogen dioxide, sulfur dioxide and carbon monoxide [Internet]. Geneva: World Health Organization; 2021. PMID: 34662007.

5. Zhang YB, Chen C, Pan XF, Guo J, Li Y, Franco OH, Liu G, Pan A. Associations of healthy lifestyle and socioeconomic status with mortality and incident cardiovascular disease: two prospective cohort studies. BMJ. 2021 Apr 14;373:n604.

6. Ye X, Wang Y, Zou Y, Tu J, Tang W, Yu R, Yang S, Huang P. Associations of socioeconomic status with infectious diseases mediated by lifestyle, environmental pollution and chronic comorbidities: a comprehensive evaluation based on UK Biobank. Infect Dis Poverty. 2023 Jan 30;12(1):5.

Table S1. Association between disease severity and risk of incident AMD.

Lung States	No. of AMD/Total sample	Risk of incident AMD			
		Model 1		Model 2	
		HR (95% CI)	*P* value	HR (95% CI)	*P* value
Normal	4235/298294	Reference	-	Reference	-
PRISm	830/48316	1.16 (1.07, 1.25)	<0.001	1.12 (1.04, 1.21)	0.004
COPD	1407/62213	1.23 (1.16, 1.31)	<0.001	1.23 (1.16, 1.31)	<0.001

Model 1 was adjusted for baseline age and sex, race, BMI, smoking and alcohol drinking status, physical activity, education level, and occupation status; model 2 was adjusted for baseline age and sex, race, BMI, smoking and alcohol drinking status, physical activity, education level, occupation status, stand height, history of hypertension, diabetes, stroke, angina, and heart attack.

Abbreviations: HR, hazards ratio; CI, confidence interval; COPD, chronic obstructive pulmonary disease; PRISm, preserved ratio impaired spirometry; AMD, age-related macular degeneration.

Table S2. Sensitivity analyses for association between incident AMD and lung function.

Lung function (Q1 vs Q4)	No. of AMD/Total sample	Risk of incident AMD			
		Model 1		Model 2	
		HR (95% CI)	*P* value	HR (95% CI)	*P* value
**Age more than 50 years old**					
FVC	2573/91764 vs 858/66275	1.22 (1.10, 1.36)	<0.001	1.18 (1.05, 1.32)	0.004
FEV1	2692/92786 vs 761/63422	1.34 (1.20, 1.48)	<0.001	1.30 (1.17, 1.45)	<0.001
PEF	2491/89574 vs 903/68352	1.34 (1.22, 1.48)	<0.001	1.31 (1.19, 1.45)	<0.001
**Adjusted for TDI additional**					
FVC	2611/103400 vs 898/101146	1.22 (1.10, 1.35)	<0.001	1.19 (1.07, 1.33)	0.002
FEV1	2728/102542 vs 809/100812	1.33 (1.20, 1.47)	<0.001	1.31 (1.17, 1.46)	<0.001
PEF	2535/102766 vs 946/101916	1.33 (1.21, 1.46)	<0.001	1.30 (1.18, 1.44)	<0.001
**Adjusted for air pollutants**					
FVC	2611/103400 vs 898/101146	1.27 (1.14, 1.41)	<0.001	1.22 (1.09, 1.37)	<0.001
FEV1	2728/102542 vs 809/100812	1.38 (1.24, 1.53)	<0.001	1.33 (1.19, 1.49)	<0.001
PEF	2535/102766 vs 946/101916	1.35 (1.23, 1.49)	<0.001	1.31 (1.19, 1.45)	<0.001
**Excluding AMD cases occurring in the first two years of follow-up**					
FVC	2478/102681 vs 858/101634	1.25 (1.12, 1.39)	<0.001	1.20 (1.07, 1.35)	0.002
FEV1	2606/101774 vs 779/101278	1.36 (1.22, 1.51)	<0.001	1.32 (1.18, 1.48)	<0.001
PEF	2414/102018 vs 905/101523	1.36 (1.23, 1.50)	<0.001	1.32 (1.20, 1.46)	<0.001

Model 1 was adjusted for baseline age and sex, race, BMI, smoking and alcohol drinking status, physical activity, education level, and occupation status; model 2 was adjusted for baseline age and sex, race, BMI, smoking and alcohol drinking status, physical activity, education level, occupation status, stand height, history of hypertension, diabetes, stroke, angina, and heart attack. Only the hazard ratios (95% confidence intervals) comparing those with the lowest lung function measures (Q1) versus those with the highest (Q4) were shown.

Abbreviations: CI, confidence interval; FEV1, forced expiratory volume in 1 second; FVC, forced vital capacity; HR, hazards ratio; PEF, peak expiratory flow; AMD, age-related macular degeneration; TDI, Townsend deprivation index.

Table S3. Association between risk for incident AMD and lung function in the White ethnicity.

Lung function	No. of AMD/Total sample	**Risk of incident AMD** (N = 386271)			
		Model 1		Model 2	
		HR (95% CI)	*P* value	HR (95% CI)	*P* value
**FVC**					
Q4	880/99593	Reference	-	Reference	-
Q3	1321/97629	1.14 (1.04, 1.24)	0.004	1.13 (1.03, 1.23)	0.010
Q2	1555/96618	1.11 (1.00, 1.22)	0.044	1.09 (0.98, 1.20)	0.117
Q1	2388/92431	1.21 (1.09, 1.34)	<0.001	1.18 (1.06, 1.33)	0.004
**FEV1**					
Q4	789/98985	Reference	-	Reference	-
Q3	1228/98248	1.13 (1.03, 1.24)	0.008	1.12 (1.02, 1.23)	0.018
Q2	1614/96996	1.19 (1.08, 1.31)	<0.001	1.17 (1.06, 1.30)	0.002
Q1	2513/92042	1.33 (1.20, 1.48)	<0.001	1.31 (1.17, 1.47)	<0.001
**PEF**					
Q4	914/97767	Reference	-	Reference	-
Q3	1236/96184	1.13 (1.03, 1.23)	0.009	1.12 (1.02, 1.22)	0.018
Q2	1611/97635	1.21 (1.10, 1.33)	<0.001	1.20 (1.08, 1.32)	<0.001
Q1	2383/94685	1.35 (1.23, 1.49)	<0.001	1.33 (1.21, 1.47)	<0.001

Model 1 was adjusted for baseline age and sex, race, BMI, smoking and alcohol drinking status, physical activity, education level, and occupation status; model 2 was adjusted for baseline age and sex, race, BMI, smoking and alcohol drinking status, physical activity, education level, occupation status, stand height, history of hypertension, diabetes, stroke, angina, and heart attack.

Abbreviations: CI, confidence interval; FEV1, forced expiratory volume in 1 second; FVC, forced vital capacity; HR, hazards ratio; PEF, peak expiratory flow; Q, quartile; AMD, age-related macular degeneration.

Table S4. Average posterior probabilities, prevalence of latent classes, and item-response probabilities in four-latent class model

Item	Latent class 1	Latent class 2	Latent class 3	Latent class 4
APP	0.97	0.98	0.99	0.99
Prevalence*	0.249	0.251	0.252	0.248
**FVC**				
Q4	0.01	0.12	0.00	**0.87**
Q3	0.14	**0.70**	0.02	0.13
Q2	**0.69**	0.18	0.13	0.00
Q1	0.16	0.00	**0.85**	0.00
**FEV1**				
Q4	0.00	0.02	0.00	**0.98**
Q3	0.01	**0.97**	0.00	0.02
Q2	**0.98**	0.01	0.01	0.00
Q1	0.01	0.00	**0.99**	0.00
**PEF**				
Q4	0.03	0.23	0.00	**0.74**
Q3	0.27	**0.46**	0.05	0.21
Q2	**0.46**	0.24	0.27	0.04
Q1	0.24	0.07	**0.68**	0.01

APP, average posterior probabilities.

* Prevalence indicated the prevalence of each latent class.

Table S5. Associations of the latent class of lung function with incident AMD

	No. of AMD/Total sample	Model 1*	Model 2**
Class 4	808/101581	Reference	Reference
Class 2	1264/102580	1.14 (1.04, 1.25)	1.12 (1.02, 1.23)
Class 1	1677/102532	1.20 (1.09, 1.32)	1.17 (1.06, 1.30)
Class 3	2728/102537	1.35 (1.21, 1.49)	1.32 (1.18, 1.47)
P for trend	-	<0.001	<0.001

* Adjusted for baseline age and sex, race, BMI, smoking and alcohol drinking status, physical activity, education level, and occupation status; ** Adjusted for baseline age and sex, race, BMI, smoking and alcohol drinking status, physical activity, education level, occupation status, stand height, and history of hypertension, diabetes, stroke, angina, and heart attack.

Table S6. Hazard ratios (95% CIs) of potential modifiers with incident AMD

**Risk factors**	**Risk of incident AMD**			
	Model 1		Model 2	
	HR (95% CI)	*P* value	HR (95% CI)	*P* value
Men	0.71 (0.67, 0.75)	<0.001	0.70 (0.65, 0.75)	<0.001
Smokers	1.16 (1.10, 1.22)	<0.001	1.14 (1.08, 1.20)	<0.001
Drinkers	1.04 (0.98, 1.10)	0.164	1.05 (1.00, 1.11)	0.060
Overweight/Obesity	1.08 (1.02, 1.14)	0.009	1.03 (0.97, 1.09)	0.366
Physical inactivity	1.07 (1.01, 1.13)	0.016	1.06 (1.00, 1.12)	0.057

Model 1 was adjusted for baseline age and sex, race, BMI, smoking and alcohol drinking status, physical activity, education level, and occupation status; model 2 was adjusted for baseline age and sex, race, BMI, smoking and alcohol drinking status, physical activity, education level, occupation status, stand height, history of hypertension, diabetes, stroke, angina, and heart attack.

Overweight/Obesity: BMI >= 25 kg/m^2; Normal weight: BMI < 25 kg/m^2.

Abbreviations: HR, hazards ratio; CI, confidence interval; FEV1, forced expiratory volume in 1 second; FVC, forced vital capacity; PEF, peak expiratory flow; AMD, age-related macular degeneration.

Table S7. Blood biomarker characteristics in the UK Biobank study.

**Blood biomarkers**	**Incident AMD**		***P* value**
**Inflammatory markers**	**No (N = 402753)**	**Yes (N = 6477)**	
Leukocyte count, 10^9 cells/L	6.85 (2.10)	6.97 (1.83)	<0.001
Neutrophil count, 10^9 cells/L	4.20 (1.39)	4.29 (1.41)	<0.001
Neutrophil percentage, %	60.8 (8.46)	61.0 (8.45)	0.023
Monocyte count, 10^9 cells/L	0.47 (0.28)	0.48 (0.19)	0.003
Monocyte percentage, %	7.06 (2.64)	7.09 (2.49)	0.309
Lymphocyte count, 10^9 cells/L	1.96 (1.16)	1.98 (0.78)	0.333
Lymphocyte percentage, %	29.0 (7.44)	28.7 (7.50)	0.003
C reactive protein, mg/L	2.48 (4.11)	3.05 (5.00)	<0.001
Platelet count, 10^9 cells/L	252 (59.4)	253 (60.7)	0.423
NLR	2.34 (1.19)	2.38 (1.17)	0.004
LMR	4.63 (3.71)	4.63 (5.37)	0.991
PLR	141 (68.0)	141 (58.4)	0.564
SII, 10^9 cells/L	593 (349)	607 (370)	0.001
**Erythrocyte-related markers**	**No (N = 402753)**	**Yes (N = 6477)**	
Erythrocyte count, 10^12 cells/L	4.52 (0.41)	4.47 (0.41)	<0.001
High light scatter reticulocyte count, 10^12 cells/L	0.02 (0.01)	0.02 (0.01)	<0.001
Reticulocyte count, 10^12 cells/L	0.06 (0.04)	0.06 (0.04)	0.162
Red blood cell (erythrocyte) distribution width, %	13.5 (0.97)	13.6 (1.06)	<0.001
Hematocrit percentage, %	41.1 (3.53)	40.7 (3.50)	<0.001
Hemoglobin concentration, g/dL	14.2 (1.24)	14.0 (1.21)	<0.001
**Metabolites**	**No (N = 97138)**	**Yes (N = 1487)**	
Total Cholesterol, mmol/L	4.58 (0.93)	4.55 (0.97)	0.213
Total Cholesterol Minus HDL-C, mmol/L	3.27 (0.83)	3.22 (0.86)	0.022
Remnant Cholesterol, mmol/L	1.55 (0.41)	1.53 (0.43)	0.042
VLDL Cholesterol, mmol/L	0.72 (0.25)	0.71 (0.25)	0.077

Clinical LDL Cholesterol, mmol/L	2.52 (0.72)	2.47 (0.75)	0.005
LDL Cholesterol, mmol/L	1.72 (0.43)	1.69 (0.45)	0.013
HDL Cholesterol, mmol/L	1.31 (0.32)	1.33 (0.33)	0.021
Total Triglycerides, mmol/L	1.29 (0.57)	1.30 (0.57)	0.444
Triglycerides in VLDL, mmol/L	0.91 (0.48)	0.91 (0.48)	0.804
Triglycerides in LDL, mmol/L	0.14 (0.04)	0.15 (0.04)	0.018
Triglycerides in HDL, mmol/L	0.14 (0.05)	0.15 (0.05)	0.002
Total Phospholipids in Lipoprotein Particles, mmol/L	2.90 (0.47)	2.91 (0.48)	0.256
Phospholipids in VLDL, mmol/L	0.47 (0.18)	0.47 (0.18)	0.391
Phospholipids in LDL, mmol/L	0.60 (0.14)	0.59 (0.14)	0.011
Phospholipids in HDL, mmol/L	1.54 (0.32)	1.57 (0.32)	<0.001
Total Esterified Cholesterol, mmol/L	3.32 (0.67)	3.30 (0.70)	0.251
Cholesteryl Esters in VLDL, mmol/L	0.43 (0.14)	0.42 (0.15)	0.034
Cholesteryl Esters in LDL, mmol/L	1.26 (0.32)	1.24 (0.33)	0.022
Cholesteryl Esters in HDL, mmol/L	1.02 (0.25)	1.03 (0.26)	0.030
Total Free Cholesterol, mmol/L	1.25 (0.27)	1.24 (0.28)	0.142
Free Cholesterol in VLDL, mmol/L	0.29 (0.11)	0.29 (0.11)	0.215
Free Cholesterol in LDL, mmol/L	0.46 (0.12)	0.45 (0.12)	0.004
Free Cholesterol in HDL, mmol/L	0.29 (0.07)	0.30 (0.07)	0.006
Total Lipids in Lipoprotein Particles, mmol/L	8.77 (1.63)	8.77 (1.67)	0.907
Total Lipids in VLDL, mmol/L	2.09 (0.85)	2.08 (0.85)	0.582
Total Lipids in LDL, mmol/L	2.46 (0.59)	2.43 (0.61)	0.025
Total Lipids in HDL, mmol/L	2.99 (0.64)	3.04 (0.64)	0.001
Total Concentration of Lipoprotein Particles, mmol/L	0.02 (<0.001)	0.02 (<0.001)	0.038
Concentration of VLDL Particles, mmol/L	<0.001 (<0.001)	<0.001 (<0.001)	0.517
Concentration of LDL Particles, mmol/L	<0.001 (<0.001)	<0.001 (<0.001)	0.007
Concentration of HDL Particles, mmol/L	0.02 (<0.001)	0.02 (<0.001)	0.010
Average Diameter for VLDL Particles, nm	38.6 (1.25)	38.6 (1.25)	0.459
Average Diameter for LDL Particles, nm	23.9 (0.09)	23.9 (0.09)	0.018
Average Diameter for HDL Particles, nm	9.65 (0.21)	9.66 (0.20)	0.017

Phosphoglycerides, mmol/L	2.26 (0.39)	2.28 (0.40)	0.019
Total Cholines, mmol/L	2.53 (0.41)	2.55 (0.41)	0.104
Phosphatidylcholines, mmol/L	2.08 (0.37)	2.10 (0.37)	0.060
Sphingomyelins, mmol/L	0.44 (0.07)	0.45 (0.07)	0.239
Apolipoprotein B, g/l	0.84 (0.20)	0.83 (0.20)	0.011
Apolipoprotein A1, g/l	1.44 (0.24)	1.46 (0.24)	0.001
Total Fatty Acids, mmol/L	11.8 (2.38)	12.0 (2.38)	0.055
Degree of Unsaturation, degree	1.36 (0.08)	1.37 (0.08)	0.011
Omega-3 Fatty Acids, mmol/L	0.53 (0.22)	0.56 (0.22)	<0.001
Omega-6 Fatty Acids, mmol/L	4.46 (0.68)	4.45 (0.67)	0.707
Polyunsaturated Fatty Acids, mmol/L	4.99 (0.80)	5.01 (0.80)	0.176
Monounsaturated Fatty Acids, mmol/L	2.81 (0.81)	2.85 (0.82)	0.056
Saturated Fatty Acids, mmol/L	4.04 (0.94)	4.09 (0.95)	0.042
Linoleic Acid, mmol/L	3.42 (0.68)	3.39 (0.68)	0.052
Docosahexaenoic Acid, mmol/L	0.23 (0.08)	0.25 (0.08)	<0.001
Alanine, mmol/L	0.30 (0.08)	0.30 (0.08)	0.405
Glutamine, mmol/L	0.53 (0.08)	0.53 (0.08)	0.453
Glycine, mmol/L	0.16 (0.06)	0.16 (0.07)	0.716
Histidine, mmol/L	0.06 (0.01)	0.06 (0.01)	0.005
Total Concentration of Branched-Chain Amino Acids, mmol/L	0.35 (0.08)	0.35 (0.09)	0.142
Isoleucine, mmol/L	0.05 (0.02)	0.05 (0.02)	0.478
Leucine, mmol/L	0.10 (0.03)	0.10 (0.03)	0.004
Valine, mmol/L	0.20 (0.04)	0.20 (0.04)	0.452
Phenylalanine, mmol/L	0.05 (0.01)	0.05 (0.01)	0.124
Tyrosine, mmol/L	0.06 (0.01)	0.06 (0.01)	<0.001
Glucose, mmol/L	3.55 (1.11)	3.75 (1.30)	<0.001
Lactate, mmol/L	3.80 (1.08)	3.79 (1.09)	0.754
Pyruvate, mmol/L	0.08 (0.03)	0.08 (0.03)	0.668
Citrate, mmol/L	0.06 (0.01)	0.06 (0.01)	<0.001

3-Hydroxybutyrate, mmol/L	0.06 (0.06)	0.06 (0.05)	0.109
Acetate, mmol/L	0.02 (0.03)	0.02 (0.04)	0.534
Acetoacetate, mmol/L	0.01 (0.01)	0.01 (0.01)	0.003
Acetone, mmol/L	0.01 (0.01)	0.01 (0.01)	0.017
Creatinine, mmol/L	0.07 (0.01)	0.07 (0.01)	0.091
Albumin, g/l	39.0 (3.28)	38.5 (3.32)	<0.001
Glycoprotein Acetyls, mmol/L	0.79 (0.11)	0.81 (0.12)	<0.001
Concentration of Chylomicrons and Extremely Large VLDL Particles, mmol/L	<0.001 (<0.001)	<0.001 (<0.001)	0.927
Total Lipids in Chylomicrons and Extremely Large VLDL, mmol/L	0.23 (0.20)	0.23 (0.20)	0.848
Phospholipids in Chylomicrons and Extremely Large VLDL, mmol/L	0.04 (0.03)	0.04 (0.03)	0.913
Cholesterol in Chylomicrons and Extremely Large VLDL, mmol/L	0.06 (0.04)	0.06 (0.04)	0.735
Cholesteryl Esters in Chylomicrons and Extremely Large VLDL, mmol/L	0.03 (0.02)	0.03 (0.02)	0.523
Free Cholesterol in Chylomicrons and Extremely Large VLDL, mmol/L	0.03 (0.02)	0.03 (0.02)	0.978
Triglycerides in Chylomicrons and Extremely Large VLDL, mmol/L	0.14 (0.13)	0.14 (0.13)	0.830
Concentration of Very Large VLDL Particles, mmol/L	<0.001 (<0.001)	<0.001 (<0.001)	0.829
Total Lipids in Very Large VLDL, mmol/L	0.20 (0.13)	0.20 (0.13)	0.764
Phospholipids in Very Large VLDL, mmol/L	0.04 (0.03)	0.04 (0.03)	0.581
Cholesterol in Very Large VLDL, mmol/L	0.05 (0.03)	0.05 (0.03)	0.197
Cholesteryl Esters in Very Large VLDL, mmol/L	0.03 (0.01)	0.03 (0.01)	0.067
Free Cholesterol in Very Large VLDL, mmol/L	0.02 (0.01)	0.02 (0.01)	0.482
Triglycerides in Very Large VLDL, mmol/L	0.11 (0.08)	0.11 (0.08)	0.892
Concentration of Large VLDL Particles, mmol/L	<0.001 (<0.001)	<0.001 (<0.001)	0.717
Total Lipids in Large VLDL, mmol/L	0.33 (0.17)	0.32 (0.17)	0.589

Phospholipids in Large VLDL, mmol/L	0.07 (0.04)	0.07 (0.04)	0.590
Cholesterol in Large VLDL, mmol/L	0.10 (0.05)	0.10 (0.05)	0.322
Cholesteryl Esters in Large VLDL, mmol/L	0.05 (0.02)	0.05 (0.02)	0.188
Free Cholesterol in Large VLDL, mmol/L	0.05 (0.02)	0.05 (0.02)	0.509
Triglycerides in Large VLDL, mmol/L	0.16 (0.09)	0.16 (0.09)	0.771
Concentration of Medium VLDL Particles, mmol/L	<0.001 (<0.001)	<0.001 (<0.001)	0.052
Total Lipids in Medium VLDL, mmol/L	0.57 (0.20)	0.56 (0.20)	0.144
Phospholipids in Medium VLDL, mmol/L	0.13 (0.05)	0.13 (0.05)	0.041
Cholesterol in Medium VLDL, mmol/L	0.17 (0.07)	0.17 (0.07)	0.002
Cholesteryl Esters in Medium VLDL, mmol/L	0.09 (0.04)	0.09 (0.04)	<0.001
Free Cholesterol in Medium VLDL, mmol/L	0.08 (0.03)	0.08 (0.03)	0.027
Triglycerides in Medium VLDL, mmol/L	0.27 (0.11)	0.27 (0.11)	0.974
Concentration of Small VLDL Particles, mmol/L	<0.001 (<0.001)	<0.001 (<0.001)	0.977
Total Lipids in Small VLDL, mmol/L	0.41 (0.13)	0.41 (0.13)	0.887
Phospholipids in Small VLDL, mmol/L	0.10 (0.03)	0.10 (0.03)	0.208
Cholesterol in Small VLDL, mmol/L	0.16 (0.05)	0.15 (0.05)	0.091
Cholesteryl Esters in Small VLDL, mmol/L	0.10 (0.03)	0.10 (0.03)	0.151
Free Cholesterol in Small VLDL, mmol/L	0.06 (0.02)	0.06 (0.02)	0.035
Triglycerides in Small VLDL, mmol/L	0.16 (0.06)	0.16 (0.06)	0.078
Concentration of Very Small VLDL Particles, mmol/L	<0.001 (<0.001)	<0.001 (<0.001)	0.877
Total Lipids in Very Small VLDL, mmol/L	0.36 (0.09)	0.36 (0.09)	0.833
Phospholipids in Very Small VLDL, mmol/L	0.10 (0.03)	0.11 (0.03)	0.668
Cholesterol in Very Small VLDL, mmol/L	0.19 (0.05)	0.18 (0.05)	0.274
Cholesteryl Esters in Very Small VLDL, mmol/L	0.13 (0.03)	0.13 (0.04)	0.144
Free Cholesterol in Very Small VLDL, mmol/L	0.06 (0.01)	0.06 (0.01)	0.863
Triglycerides in Very Small VLDL, mmol/L	0.07 (0.02)	0.07 (0.02)	0.004
Concentration of IDL Particles, mmol/L	<0.001 (<0.001)	<0.001 (<0.001)	0.011
Total Lipids in IDL, mmol/L	1.23 (0.29)	1.22 (0.30)	0.133
Phospholipids in IDL, mmol/L	0.29 (0.07)	0.29 (0.07)	0.123
Cholesterol in IDL, mmol/L	0.84 (0.21)	0.83 (0.23)	0.057

Cholesteryl Esters in IDL, mmol/L	0.62 (0.16)	0.61 (0.17)	0.044
Free Cholesterol in IDL, mmol/L	0.22 (0.06)	0.22 (0.06)	0.118
Triglycerides in IDL, mmol/L	0.10 (0.03)	0.10 (0.03)	0.004
Concentration of Large LDL Particles, mmol/L	<0.001 (<0.001)	<0.001 (<0.001)	0.004
Total Lipids in Large LDL, mmol/L	1.57 (0.37)	1.55 (0.38)	0.035
Phospholipids in Large LDL, mmol/L	0.35 (0.08)	0.35 (0.08)	0.014
Cholesterol in Large LDL, mmol/L	1.12 (0.28)	1.10 (0.29)	0.019
Cholesteryl Esters in Large LDL, mmol/L	0.82 (0.20)	0.81 (0.21)	0.027
Free Cholesterol in Large LDL, mmol/L	0.29 (0.08)	0.29 (0.08)	0.009
Triglycerides in Large LDL, mmol/L	0.10 (0.03)	0.10 (0.02)	0.007
Concentration of Medium LDL Particles, mmol/L	<0.001 (<0.001)	<0.001 (<0.001)	0.027
Total Lipids in Medium LDL, mmol/L	0.61 (0.16)	0.60 (0.16)	0.025
Phospholipids in Medium LDL, mmol/L	0.16 (0.04)	0.16 (0.04)	0.012
Cholesterol in Medium LDL, mmol/L	0.42 (0.12)	0.41 (0.12)	0.015
Cholesteryl Esters in Medium LDL, mmol/L	0.30 (0.09)	0.30 (0.09)	0.030
Free Cholesterol in Medium LDL, mmol/L	0.12 (0.03)	0.12 (0.03)	0.003
Triglycerides in Medium LDL, mmol/L	0.03 (0.01)	0.03 (0.01)	0.044
Concentration of Small LDL Particles, mmol/L	<0.001 (<0.001)	<0.001 (<0.001)	0.017
Total Lipids in Small LDL, mmol/L	0.28 (0.06)	0.28 (0.07)	0.008
Phospholipids in Small LDL, mmol/L	0.09 (0.02)	0.09 (0.02)	0.011
Cholesterol in Small LDL, mmol/L	0.18 (0.04)	0.18 (0.04)	0.003
Cholesteryl Esters in Small LDL, mmol/L	0.13 (0.03)	0.13 (0.03)	0.008
Free Cholesterol in Small LDL, mmol/L	0.05 (0.01)	0.05 (0.01)	<0.001
Triglycerides in Small LDL, mmol/L	0.02 (0.01)	0.02 (0.01)	0.237
Concentration of Very Large HDL Particles, mmol/L	<0.001 (<0.001)	<0.001 (<0.001)	0.244
Total Lipids in Very Large HDL, mmol/L	0.17 (0.08)	0.17 (0.08)	0.237
Phospholipids in Very Large HDL, mmol/L	0.08 (0.04)	0.08 (0.05)	0.149
Cholesterol in Very Large HDL, mmol/L	0.08 (0.03)	0.08 (0.04)	0.504
Cholesteryl Esters in Very Large HDL, mmol/L	0.06 (0.03)	0.06 (0.03)	0.410
Free Cholesterol in Very Large HDL, mmol/L	0.02 (0.01)	0.02 (0.01)	0.963

Triglycerides in Very Large HDL, mmol/L	0.01 (<0.001)	0.01 (<0.001)	0.013
Concentration of Large HDL Particles, mmol/L	<0.001 (<0.001)	<0.001 (<0.001)	0.028
Total Lipids in Large HDL, mmol/L	0.65 (0.32)	0.67 (0.33)	0.017
Phospholipids in Large HDL, mmol/L	0.32 (0.15)	0.33 (0.15)	0.005
Cholesterol in Large HDL, mmol/L	0.30 (0.17)	0.30 (0.17)	0.078
Cholesteryl Esters in Large HDL, mmol/L	0.23 (0.13)	0.23 (0.13)	0.093
Free Cholesterol in Large HDL, mmol/L	0.07 (0.04)	0.07 (0.04)	0.041
Triglycerides in Large HDL, mmol/L	0.03 (0.01)	0.03 (0.01)	<0.001
Concentration of Medium HDL Particles, mmol/L	<0.001 (<0.001)	<0.001 (<0.001)	<0.001
Total Lipids in Medium HDL, mmol/L	1.03 (0.22)	1.05 (0.22)	<0.001
Phospholipids in Medium HDL, mmol/L	0.48 (0.10)	0.49 (0.10)	<0.001
Cholesterol in Medium HDL, mmol/L	0.49 (0.12)	0.50 (0.12)	0.002
Cholesteryl Esters in Medium HDL, mmol/L	0.40 (0.10)	0.41 (0.10)	0.003
Free Cholesterol in Medium HDL, mmol/L	0.09 (0.02)	0.09 (0.02)	<0.001
Triglycerides in Medium HDL, mmol/L	0.05 (0.02)	0.06 (0.02)	0.002
Concentration of Small HDL Particles, mmol/L	0.01 (<0.001)	0.01 (<0.001)	0.353
Total Lipids in Small HDL, mmol/L	1.15 (0.16)	1.16 (0.16)	0.027
Phospholipids in Small HDL, mmol/L	0.66 (0.09)	0.66 (0.09)	0.005
Cholesterol in Small HDL, mmol/L	0.44 (0.06)	0.44 (0.06)	0.345
Cholesteryl Esters in Small HDL, mmol/L	0.33 (0.05)	0.33 (0.05)	0.712
Free Cholesterol in Small HDL, mmol/L	0.11 (0.02)	0.11 (0.02)	0.013
Triglycerides in Small HDL, mmol/L	0.05 (0.02)	0.05 (0.02)	0.058

Data are presented as mean (SD). P values were calculated using t-test.

Abbreviations: HDL, high density lipoprotein; LDL, low density lipoprotein; LMR, lymphocyte-to-monocyte ratio; NLR, neutrophil-to-lymphocyte ratio; PLR, platelet-to-lymphocyte ratio; SII, systemic immune-inflammation index; VLDL, very low-density lipoprotein.

Table S8. Associations between lung function measures with inflammatory and erythrocyte-related markers at baseline.

Blood biomarkers		Decrements in lung function measures					
		FVC		FEV1		PEF	
**Inflammatory markers (mean [SD])**		**Beta**	***P* value FDR**	**Beta**	***P* value FDR**	**Beta**	***P* value FDR**
Leukocyte count, 10^9 cells/L	6.85 (2.10)	0.034	<0.001	0.040	<0.001	5.140	<0.001
Neutrophil count, 10^9 cells/L	4.20 (1.39)	0.034	<0.001	0.041	<0.001	6.089	<0.001
Neutrophil percentage, %	60.8 (8.46)	0.003	0.001	0.008	<0.001	2.575	<0.001
Monocyte count, 10^9 cells/L	0.47 (0.28)	0.024	<0.001	0.023	<0.001	2.586	<0.001
Monocyte percentage, %	7.06 (2.64)	0.008	<0.001	0.004	<0.001	0.005	0.976
Lymphocyte count, 10^9 cells/L	1.96 (1.16)	0.012	<0.001	0.011	<0.001	0.762	<0.001
Lymphocyte percentage, %	29.0 (7.44)	-0.011	<0.001	-0.017	<0.001	-3.731	<0.001
C reactive protein, mg/L	2.49 (4.13)	0.048	<0.001	0.046	<0.001	6.561	<0.001
Platelet count, 10^9 cells/L	252 (59.4)	0.006	<0.001	0.010	<0.001	2.132	<0.001
NLR	2.34 (1.19)	0.012	<0.001	0.017	<0.001	3.528	<0.001
LMR	4.63 (3.74)	-0.005	<0.001	-0.004	<0.001	-0.463	0.002
PLR	141 (67.9)	-0.007	<0.001	-0.005	<0.001	0.818	<0.001
SII, 10^9 cells/L	593 (350)	0.014	<0.001	0.019	<0.001	3.967	<0.001

Erythrocyte-related markers (mean [SD])		Beta	*P* value FDR	Beta	*P* value FDR	Beta	*P* value FDR
Erythrocyte count, 10^12 cells/L	4.52 (0.41)	0.006	<0.001	-0.002	0.082	-2.507	<0.001
High light scatter reticulocyte count, 10^12 cells/L	0.02 (0.01)	0.037	<0.001	0.029	<0.001	2.742	<0.001
Reticulocyte count, 10^12 cells/L	0.06 (0.04)	0.016	<0.001	0.012	<0.001	0.748	<0.001
Red blood cell distribution width, %	13.5 (0.97)	0.023	<0.001	0.025	<0.001	3.584	<0.001
Hematocrit percentage, %	41.1 (3.53)	0.004	<0.001	0.002	0.038	-1.943	<0.001
Hemoglobin concentration, g/dL	14.2 (1.24)	-0.005	<0.001	-0.005	<0.001	-2.446	<0.001

Lung function data were initially minus-transformed. The standardized coefficients were provided. False discovery rate (FDR) adjusted *P* values < 0.05 indicate statistically associations, as determined based on multiple-adjusted linear models (lung function as independent variables, blood metabolites as dependent variables). Multivariate linear regression models were adjusted for baseline age and sex, race, BMI, smoking and alcohol drinking status, physical activity, education level, occupation status, stand height, history of hypertension, diabetes, stroke, angina, and heart attack.

Abbreviations: FEV1, forced expiratory volume in 1 second; FVC, forced vital capacity; LMR, lymphocyte-to-monocyte ratio; NLR, neutrophil-to-lymphocyte ratio; PEF, peak expiratory flow; PLR, platelet-to-lymphocyte ratio; SII, systemic immune-inflammation index.

Table S9. Associations between lung function measures with metabolites at baseline.

Metabolites (mean [SD])		Decrements in lung function measures					
		FVC		FEV1		PEF	
		Beta	*P* value FDR	Beta	*P* value FDR	Beta	*P* value FDR
Total Cholesterol, mmol/L	4.58 (0.93)	-0.011	<0.001	-0.014	<0.001	-2.318	<0.001
Total Cholesterol Minus HDL-C, mmol/L	3.27 (0.83)	-0.005	0.028	-0.011	<0.001	-2.051	<0.001
Remnant Cholesterol, mmol/L	1.55 (0.41)	-0.001	0.659	-0.008	<0.001	-1.487	<0.001
VLDL Cholesterol, mmol/L	0.72 (0.25)	0.007	<0.001	-0.002	0.332	-0.643	0.047
Clinical LDL Cholesterol, mmol/L	2.52 (0.72)	-0.010	<0.001	-0.015	<0.001	-2.662	<0.001
LDL Cholesterol, mmol/L	1.72 (0.43)	-0.008	<0.001	-0.013	<0.001	-2.502	<0.001
HDL Cholesterol, mmol/L	1.31 (0.32)	-0.023	<0.001	-0.012	<0.001	-1.272	<0.001
Total Triglycerides, mmol/L	1.29 (0.57)	0.026	<0.001	0.016	<0.001	1.712	<0.001
Triglycerides in VLDL, mmol/L	0.91 (0.48)	0.026	<0.001	0.015	<0.001	1.499	<0.001
Triglycerides in LDL, mmol/L	0.14 (0.04)	0.028	<0.001	0.019	<0.001	2.340	<0.001
Triglycerides in HDL, mmol/L	0.14 (0.05)	0.021	<0.001	0.016	<0.001	2.035	<0.001
Total Phospholipids in Lipoprotein Particles, mmol/L	2.90 (0.47)	-0.001	0.738	-0.002	0.169	-0.699	0.040
Phospholipids in VLDL, mmol/L	0.47 (0.18)	0.016	<0.001	0.006	<0.001	0.363	0.285
Phospholipids in LDL, mmol/L	0.60 (0.14)	-0.005	0.011	-0.012	<0.001	-2.243	<0.001
Phospholipids in HDL, mmol/L	1.54 (0.32)	-0.009	<0.001	<0.001	0.926	0.189	0.629
Total Esterified Cholesterol, mmol/L	3.32 (0.67)	-0.012	<0.001	-0.015	<0.001	-2.452	<0.001
Cholesteryl Esters in VLDL, mmol/L	0.43 (0.14)	0.003	0.141	-0.005	0.005	-1.001	0.002
Cholesteryl Esters in LDL, mmol/L	1.26 (0.32)	-0.005	0.008	-0.012	<0.001	-2.291	<0.001

Cholesteryl Esters in HDL, mmol/L	1.02 (0.25)	-0.025	<0.001	-0.014	<0.001	-1.539	<0.001
Total Free Cholesterol, mmol/L	1.25 (0.27)	-0.007	0.001	-0.011	<0.001	-1.949	<0.001
Free Cholesterol in VLDL, mmol/L	0.29 (0.11)	0.012	<0.001	0.003	0.143	-0.145	0.675
Free Cholesterol in LDL, mmol/L	0.46 (0.12)	-0.014	<0.001	-0.018	<0.001	-2.991	<0.001
Free Cholesterol in HDL, mmol/L	0.29 (0.07)	-0.013	<0.001	-0.005	0.018	-0.276	0.493
Total Lipids in Lipoprotein Particles, mmol/L	8.77 (1.63)	0.003	0.145	-0.002	0.164	-0.837	0.010
Total Lipids in VLDL, mmol/L	2.09 (0.85)	0.020	<0.001	0.009	<0.001	0.704	0.032
Total Lipids in LDL, mmol/L	2.46 (0.59)	-0.005	0.017	-0.011	<0.001	-2.187	<0.001
Total Lipids in HDL, mmol/L	2.99 (0.64)	-0.014	<0.001	-0.004	0.0374	-0.327	0.399
Total Concentration of Lipoprotein Particles, mmol/L	0.02 (<0.001)	-0.015	<0.001	-0.010	<0.001	-1.876	<0.001
Concentration of VLDL Particles, mmol/L	<0.001 (<0.001)	0.012	<0.001	0.003	0.112	-0.090	0.802
Concentration of LDL Particles, mmol/L	<0.001 (<0.001)	-0.003	0.230	-0.010	<0.001	-1.860	<0.001
Concentration of HDL Particles, mmol/L	0.02 (<0.001)	-0.016	<0.001	-0.009	<0.001	-1.654	<0.001
Average Diameter for VLDL Particles, nm	38.6 (1.25)	0.020	<0.001	0.009	<0.001	0.516	0.149
Average Diameter for LDL Particles, nm	23.9 (0.09)	-0.018	<0.001	-0.012	<0.001	-1.479	<0.001
Average Diameter for HDL Particles, nm	9.65 (0.21)	-0.018	<0.001	-0.005	0.020	0.654	0.085
Phosphoglycerides, mmol/L	2.26 (0.39)	<0.001	0.966	<0.001	0.839	-0.401	0.256
Total Cholines, mmol/L	2.54 (0.41)	-0.004	0.046	-0.004	0.025	-0.868	0.011
Phosphatidylcholines, mmol/L	2.08 (0.37)	-0.004	0.106	-0.003	0.132	-0.757	0.028
Sphingomyelins, mmol/L	0.44 (0.07)	-0.007	0.001	-0.007	<0.001	-1.065	0.002
Apolipoprotein B, g/l	0.84 (0.20)	-0.001	0.665	-0.009	<0.001	-1.682	<0.001
Apolipoprotein A1, g/l	1.44 (0.24)	-0.013	<0.001	-0.005	0.008	-0.685	0.059
Total Fatty Acids, mmol/L	11.9 (2.38)	0.016	<0.001	0.009	<0.001	0.864	0.007

Degree of Unsaturation, degree	1.36 (0.08)	-0.034	<0.001	-0.035	<0.001	-6.085	<0.001
Omega-3 Fatty Acids, mmol/L	0.53 (0.22)	-0.004	0.092	-0.013	<0.001	-3.194	<0.001
Omega-6 Fatty Acids, mmol/L	4.46 (0.68)	-0.003	0.122	-0.007	<0.001	-1.407	<0.001
Polyunsaturated Fatty Acids, mmol/L	4.99 (0.80)	-0.004	0.074	-0.009	<0.001	-2.098	<0.001
Monounsaturated Fatty Acids, mmol/L	2.81 (0.81)	0.025	<0.001	0.019	<0.001	2.476	<0.001
Saturated Fatty Acids, mmol/L	4.04 (0.94)	0.021	<0.001	0.015	<0.001	1.841	<0.001
Linoleic Acid, mmol/L	3.42 (0.68)	-0.005	0.010	-0.008	<0.001	-1.589	<0.001
Docosahexaenoic Acid, mmol/L	0.23 (0.08)	-0.015	<0.001	-0.022	<0.001	-4.379	<0.001
Alanine, mmol/L	0.30 (0.08)	0.005	0.016	-0.002	0.291	-2.008	<0.001
Glutamine, mmol/L	0.53 (0.08)	-0.014	<0.001	-0.01	<0.001	-0.351	0.285
Glycine, mmol/L	0.16 (0.06)	-0.016	<0.001	-0.01	<0.001	-0.040	0.915
Histidine, mmol/L	0.06 (0.01)	-0.011	<0.001	-0.014	<0.001	-2.649	<0.001
Total Concentration of Branched-Chain Amino Acids, mmol/L	0.35 (0.08)	0.012	<0.001	0.005	0.004	-0.855	0.009
Isoleucine, mmol/L	0.05 (0.02)	0.013	<0.001	0.008	<0.001	0.312	0.351
Leucine, mmol/L	0.10 (0.03)	0.007	0.001	0.002	0.255	-1.099	<0.001
Valine, mmol/L	0.20 (0.04)	0.015	<0.001	0.005	0.004	-1.147	<0.001
Phenylalanine, mmol/L	0.05 (0.01)	0.016	<0.001	0.014	<0.001	1.210	<0.001
Tyrosine, mmol/L	0.06 (0.01)	0.026	<0.001	0.020	<0.001	1.030	0.002
Glucose, mmol/L	3.56 (1.11)	0.009	<0.001	0.003	0.086	0.751	0.028
Lactate, mmol/L	3.80 (1.08)	0.011	<0.001	0.007	<0.001	-1.278	<0.001
Pyruvate, mmol/L	0.08 (0.03)	0.005	0.010	0.004	0.018	-0.151	0.650
Citrate, mmol/L	0.06 (0.01)	-0.019	<0.001	-0.018	<0.001	-2.362	<0.001
3-Hydroxybutyrate, mmol/L	0.06 (0.06)	-0.014	<0.001	-0.007	<0.001	0.136	0.680
Acetate, mmol/L	0.02 (0.03)	-0.003	0.201	0.002	0.247	1.559	<0.001
Acetoacetate, mmol/L	0.01 (0.01)	-0.007	<0.001	-0.001	0.673	1.561	<0.001
Acetone, mmol/L	0.01 (0.01)	-0.014	<0.001	-0.008	<0.001	-0.143	0.669

Creatinine, mmol/L	0.07 (0.01)	-0.006	0.008	-0.006	0.001	-1.831	<0.001
Albumin, g/l	39.0 (3.28)	-0.031	<0.001	-0.035	<0.001	-5.359	<0.001
Glycoprotein Acetyls, mmol/L	0.79 (0.11)	0.049	<0.001	0.043	<0.001	5.884	<0.001
Concentration of Chylomicrons and Extremely Large VLDL Particles, mmol/L	<0.001 (<0.001)	0.029	<0.001	0.019	<0.001	2.442	<0.001
Total Lipids in Chylomicrons and Extremely Large VLDL, mmol/L	0.23 (0.20)	0.029	<0.001	0.019	<0.001	2.466	<0.001
Phospholipids in Chylomicrons and Extremely Large VLDL, mmol/L	0.04 (0.03)	0.028	<0.001	0.019	<0.001	2.357	<0.001
Cholesterol in Chylomicrons and Extremely Large VLDL, mmol/L	0.06 (0.04)	0.025	<0.001	0.015	<0.001	1.909	<0.001
Cholesteryl Esters in Chylomicrons and Extremely Large VLDL, mmol/L	0.03 (0.02)	0.023	<0.001	0.014	<0.001	1.694	<0.001
Free Cholesterol in Chylomicrons and Extremely Large VLDL, mmol/L	0.03 (0.02)	0.026	<0.001	0.017	<0.001	2.142	<0.001
Triglycerides in Chylomicrons and Extremely Large VLDL, mmol/L	0.14 (0.13)	0.030	<0.001	0.020	<0.001	2.631	<0.001
Concentration of Very Large VLDL Particles, mmol/L	<0.001 (<0.001)	0.026	<0.001	0.015	<0.001	1.469	<0.001
Total Lipids in Very Large VLDL, mmol/L	0.20 (0.13)	0.026	<0.001	0.015	<0.001	1.528	<0.001
Phospholipids in Very Large VLDL, mmol/L	0.04 (0.03)	0.025	<0.001	0.013	<0.001	1.373	<0.001
Cholesterol in Very Large VLDL, mmol/L	0.05 (0.03)	0.018	<0.001	0.007	<0.001	0.442	0.196

Cholesteryl Esters in Very Large VLDL, mmol/L	0.03 (0.01)	0.013	<0.001	0.002	0.182	-0.208	0.560
Free Cholesterol in Very Large VLDL, mmol/L	0.02 (0.01)	0.022	<0.001	0.011	<0.001	1.102	0.002
Triglycerides in Very Large VLDL, mmol/L	0.11 (0.08)	0.029	<0.001	0.018	<0.001	1.923	<0.001
Concentration of Large VLDL Particles, mmol/L	<0.001 (<0.001)	0.022	<0.001	0.011	<0.001	0.813	0.014
Total Lipids in Large VLDL, mmol/L	0.33 (0.17)	0.021	<0.001	0.010	<0.001	0.624	0.061
Phospholipids in Large VLDL, mmol/L	0.07 (0.04)	0.023	<0.001	0.011	<0.001	0.922	0.006
Cholesterol in Large VLDL, mmol/L	0.10 (0.05)	0.015	<0.001	0.004	0.019	-0.054	0.883
Cholesteryl Esters in Large VLDL, mmol/L	0.05 (0.02)	0.009	<0.001	-0.001	0.770	-0.639	0.050
Free Cholesterol in Large VLDL, mmol/L	0.05 (0.02)	0.020	<0.001	0.009	<0.001	0.524	0.123
Triglycerides in Large VLDL, mmol/L	0.16 (0.09)	0.023	<0.001	0.012	<0.001	0.830	0.013
Concentration of Medium VLDL Particles, mmol/L	<0.001 (<0.001)	0.004	0.089	-0.005	0.001	-1.336	<0.001
Total Lipids in Medium VLDL, mmol/L	0.57 (0.20)	0.008	<0.001	-0.002	0.328	-0.939	0.003
Phospholipids in Medium VLDL, mmol/L	0.13 (0.05)	0.004	0.081	-0.005	0.003	-1.277	<0.001
Cholesterol in Medium VLDL, mmol/L	0.17 (0.07)	-0.008	<0.001	-0.014	<0.001	-2.326	<0.001
Cholesteryl Esters in Medium VLDL, mmol/L	0.09 (0.04)	-0.014	<0.001	-0.018	<0.001	-2.743	<0.001
Free Cholesterol in Medium VLDL, mmol/L	0.08 (0.03)	<0.001	0.864	-0.008	<0.001	-1.678	<0.001
Triglycerides in Medium VLDL, mmol/L	0.27 (0.11)	0.018	<0.001	0.007	<0.001	0.172	0.628

Concentration of Small VLDL Particles, mmol/L	<0.001 (<0.001)	0.013	<0.001	0.003	0.060	-0.083	0.813
Total Lipids in Small VLDL, mmol/L	0.41 (0.13)	0.012	<0.001	0.003	0.120	-0.198	0.572
Phospholipids in Small VLDL, mmol/L	0.10 (0.03)	0.005	0.010	-0.004	0.034	-1.075	<0.001
Cholesterol in Small VLDL, mmol/L	0.16 (0.05)	0.003	0.141	-0.005	0.001	-1.192	<0.001
Cholesteryl Esters in Small VLDL, mmol/L	0.10 (0.03)	0.005	0.013	-0.004	0.040	-0.892	0.006
Free Cholesterol in Small VLDL, mmol/L	0.06 (0.02)	-0.001	0.793	-0.008	<0.001	-1.698	<0.001
Triglycerides in Small VLDL, mmol/L	0.16 (0.06)	0.022	<0.001	0.013	<0.001	1.166	<0.001
Concentration of Very Small VLDL Particles, mmol/L	<0.001 (<0.001)	0.009	<0.001	0.002	0.162	0.196	0.574
Total Lipids in Very Small VLDL, mmol/L	0.36 (0.09)	0.010	<0.001	0.004	0.032	0.406	0.225
Phospholipids in Very Small VLDL, mmol/L	0.10 (0.03)	0.013	<0.001	0.006	<0.001	0.763	0.018
Cholesterol in Very Small VLDL, mmol/L	0.19 (0.05)	-0.001	0.806	-0.005	0.006	-0.712	0.032
Cholesteryl Esters in Very Small VLDL, mmol/L	0.13 (0.03)	-0.004	0.077	-0.007	<0.001	-1.012	0.003
Free Cholesterol in Very Small VLDL, mmol/L	0.06 (0.01)	0.007	0.001	0.001	0.738	-0.020	0.958
Triglycerides in Very Small VLDL, mmol/L	0.07 (0.02)	0.026	<0.001	0.018	<0.001	2.281	<0.001
Concentration of IDL Particles, mmol/L	<0.001 (<0.001)	-0.003	0.152	-0.009	<0.001	-1.674	<0.001
Total Lipids in IDL, mmol/L	1.23 (0.29)	-0.007	0.001	-0.010	<0.001	-1.810	<0.001
Phospholipids in IDL, mmol/L	0.29 (0.07)	-0.006	0.003	-0.010	<0.001	-1.594	<0.001

Cholesterol in IDL, mmol/L	0.84 (0.21)	-0.011	<0.001	-0.014	<0.001	-2.309	<0.001
Cholesteryl Esters in IDL, mmol/L	0.62 (0.16)	-0.011	<0.001	-0.013	<0.001	-2.273	<0.001
Free Cholesterol in IDL, mmol/L	0.22 (0.06)	-0.012	<0.001	-0.015	<0.001	-2.343	<0.001
Triglycerides in IDL, mmol/L	0.10 (0.03)	0.027	<0.001	0.019	<0.001	2.501	<0.001
Concentration of Large LDL Particles, mmol/L	<0.001 (<0.001)	-0.004	0.036	-0.011	<0.001	-1.905	<0.001
Total Lipids in Large LDL, mmol/L	1.57 (0.37)	-0.008	<0.001	-0.013	<0.001	-2.353	<0.001
Phospholipids in Large LDL, mmol/L	0.35 (0.08)	-0.009	<0.001	-0.014	<0.001	-2.479	<0.001
Cholesterol in Large LDL, mmol/L	1.12 (0.28)	-0.010	<0.001	-0.015	<0.001	-2.651	<0.001
Cholesteryl Esters in Large LDL, mmol/L	0.82 (0.20)	-0.009	<0.001	-0.014	<0.001	-2.500	<0.001
Free Cholesterol in Large LDL, mmol/L	0.29 (0.08)	-0.015	<0.001	-0.018	<0.001	-3.001	<0.001
Triglycerides in Large LDL, mmol/L	0.10 (0.03)	0.028	<0.001	0.019	<0.001	2.405	<0.001
Concentration of Medium LDL Particles, mmol/L	<0.001 (<0.001)	-0.001	0.793	-0.009	<0.001	-1.835	<0.001
Total Lipids in Medium LDL, mmol/L	0.61 (0.16)	-0.001	0.700	-0.009	<0.001	-1.960	<0.001
Phospholipids in Medium LDL, mmol/L	0.16 (0.04)	-0.001	0.500	-0.009	<0.001	-2.059	<0.001
Cholesterol in Medium LDL, mmol/L	0.42 (0.12)	-0.003	0.132	-0.011	<0.001	-2.212	<0.001
Cholesteryl Esters in Medium LDL, mmol/L	0.30 (0.09)	<0.001	0.953	-0.008	<0.001	-1.891	<0.001
Free Cholesterol in Medium LDL, mmol/L	0.12 (0.03)	-0.011	<0.001	-0.016	<0.001	-2.943	<0.001
Triglycerides in Medium LDL, mmol/L	0.03 (0.01)	0.028	<0.001	0.018	<0.001	2.184	<0.001
Concentration of Small LDL Particles, mmol/L	<0.001 (<0.001)	0.003	0.167	-0.006	<0.001	-1.323	<0.001
Total Lipids in Small LDL, mmol/L	0.28 (0.06)	0.001	0.693	-0.007	<0.001	-1.646	<0.001
Phospholipids in Small LDL, mmol/L	0.09 (0.02)	0.002	0.451	-0.006	<0.001	-1.403	<0.001

Cholesterol in Small LDL, mmol/L	0.18 (0.04)	-0.003	0.172	-0.010	<0.001	-2.104	<0.001
Cholesteryl Esters in Small LDL, mmol/L	0.13 (0.03)	<0.001	0.914	-0.008	<0.001	-1.745	<0.001
Free Cholesterol in Small LDL, mmol/L	0.05 (0.01)	-0.011	<0.001	-0.016	<0.001	-2.826	<0.001
Triglycerides in Small LDL, mmol/L	0.02 (0.01)	0.028	<0.001	0.018	<0.001	2.106	<0.001
Concentration of Very Large HDL Particles, mmol/L	<0.001 (<0.001)	-0.012	<0.001	-0.005	0.006	0.370	0.333
Total Lipids in Very Large HDL, mmol/L	0.17 (0.08)	-0.012	<0.001	-0.005	0.018	0.521	0.166
Phospholipids in Very Large HDL, mmol/L	0.08 (0.04)	-0.011	<0.001	-0.003	0.094	0.745	0.043
Cholesterol in Very Large HDL, mmol/L	0.08 (0.03)	-0.016	<0.001	-0.008	<0.001	0.019	0.962
Cholesteryl Esters in Very Large HDL, mmol/L	0.06 (0.03)	-0.018	<0.001	-0.009	<0.001	-0.244	0.549
Free Cholesterol in Very Large HDL, mmol/L	0.02 (0.01)	-0.007	0.004	-0.002	0.369	0.927	0.009
Triglycerides in Very Large HDL, mmol/L	0.01 (<0.001)	0.018	<0.001	0.013	<0.001	2.056	<0.001
Concentration of Large HDL Particles, mmol/L	<0.001 (<0.001)	-0.021	<0.001	-0.009	<0.001	-0.377	0.342
Total Lipids in Large HDL, mmol/L	0.65 (0.32)	-0.018	<0.001	-0.007	<0.001	-0.088	0.828
Phospholipids in Large HDL, mmol/L	0.32 (0.15)	-0.015	<0.001	-0.004	0.049	0.245	0.549
Cholesterol in Large HDL, mmol/L	0.30 (0.17)	-0.023	<0.001	-0.011	<0.001	-0.573	0.139
Cholesteryl Esters in Large HDL, mmol/L	0.23 (0.13)	-0.024	<0.001	-0.012	<0.001	-0.767	0.043
Free Cholesterol in Large HDL, mmol/L	0.07 (0.04)	-0.016	<0.001	-0.006	0.003	0.104	0.802
Triglycerides in Large HDL, mmol/L	0.03 (0.01)	0.010	<0.001	0.010	<0.001	1.760	<0.001

Concentration of Medium HDL Particles, mmol/L	<0.001 (<0.001)	-0.013	<0.001	-0.004	0.035	-0.593	0.102
Total Lipids in Medium HDL, mmol/L	1.03 (0.22)	-0.009	<0.001	-0.001	0.741	-0.195	0.611
Phospholipids in Medium HDL, mmol/L	0.48 (0.10)	-0.004	0.077	0.003	0.082	0.277	0.458
Cholesterol in Medium HDL, mmol/L	0.49 (0.12)	-0.018	<0.001	-0.007	<0.001	-1.008	0.004
Cholesteryl Esters in Medium HDL, mmol/L	0.40 (0.10)	-0.019	<0.001	-0.008	<0.001	-1.132	0.002
Free Cholesterol in Medium HDL, mmol/L	0.09 (0.02)	-0.012	<0.001	-0.003	0.083	-0.468	0.211
Triglycerides in Medium HDL, mmol/L	0.05 (0.02)	0.020	<0.001	0.016	<0.001	1.992	<0.001
Concentration of Small HDL Particles, mmol/L	0.01 (<0.001)	-0.008	<0.001	-0.008	<0.001	-2.130	<0.001
Total Lipids in Small HDL, mmol/L	1.15 (0.16)	0.001	0.714	<0.001	0.852	-0.881	0.006
Phospholipids in Small HDL, mmol/L	0.66 (0.09)	0.003	0.127	0.004	0.039	-0.331	0.333
Cholesterol in Small HDL, mmol/L	0.44 (0.06)	-0.010	<0.001	-0.009	<0.001	-2.287	<0.001
Cholesteryl Esters in Small HDL, mmol/L	0.33 (0.05)	-0.013	<0.001	-0.012	<0.001	-2.608	<0.001
Free Cholesterol in Small HDL, mmol/L	0.11 (0.02)	<0.001	0.966	-0.001	0.498	-0.939	0.004
Triglycerides in Small HDL, mmol/L	0.05 (0.02)	0.027	<0.001	0.018	<0.001	1.993	<0.001

Lung function data were initially minus-transformed. The standardized coefficients were thus provided. False discovery rate (FDR) adjusted *P* values < 0.05 indicate statistically associations, as determined based on multiple-adjusted linear models (lung function as independent variables, blood metabolites as dependent variables). Multivariate linear regression models were adjusted for baseline age and sex, race, BMI, smoking and alcohol drinking status, physical activity, education level, occupation status, stand height, history of hypertension, diabetes, stroke, angina, and heart attack.

Abbreviations: FEV1, forced expiratory volume in 1 second; FVC, forced vital capacity; HDL, high density lipoprotein; LDL, low density lipoprotein; PEF, peak expiratory flow. VLDL, very low-density lipoprotein.

Table S10. Longitudinal associations between the risk of incident AMD with inflammatory and erythrocyte-related markers.

Blood biomarkers		Incident AMD			
**Inflammatory markers (mean [SD])**		**HR**	**95% CI**	***P* value**	***P* value FDR**
Leukocyte count, 10^9 cells/L	6.85 (2.10)	1.02	1.00-1.04	0.103	0.222
Neutrophil count, 10^9 cells/L	4.20 (1.39)	1.04	1.02-1.07	0.002	0.039
Neutrophil percentage, %	60.8 (8.46)	1.03	1.01-1.06	0.008	0.107
Monocyte count, 10^9 cells/L	0.47 (0.28)	1.01	0.98-1.03	0.697	0.852
Monocyte percentage, %	7.06 (2.64)	1.00	0.97-1.02	0.729	0.870
Lymphocyte count, 10^9 cells/L	1.96 (1.16)	0.98	0.94-1.01	0.196	0.336
Lymphocyte percentage, %	29.0 (7.44)	0.96	0.93-0.98	0.001	0.035
C reactive protein, mg/L	2.49 (4.13)	1.06	1.03-1.08	<0.001	<0.001
Platelet count, 10^9 cells/L	252 (59.4)	1.02	0.99-1.04	0.211	0.344
NLR	2.34 (1.19)	1.03	1.01-1.05	0.003	0.068
LMR	4.63 (3.74)	1.00	0.98-1.03	0.779	0.899
PLR	141 (67.9)	1.01	1.00-1.02	0.177	0.312
SII, 10^9 cells/L	593 (350)	1.03	1.01-1.05	<0.001	0.021
**Erythrocyte-related markers (mean [SD])**		**HR**	**95% CI**	***P* value**	***P* value FDR**
Erythrocyte count, 10^12 cells/L	4.52 (0.41)	0.95	0.93-0.98	0.001	0.035
High light scatter reticulocyte count, 10^12 cells/L	0.02 (0.01)	1.01	0.98-1.04	0.454	0.615
Reticulocyte count, 10^12 cells/L	0.06 (0.04)	1.01	0.99-1.03	0.460	0.619

Red blood cell distribution width, %	13.5 (0.97)	1.08	1.05-1.10	<0.001	<0.001
Hematocrit percentage, %	41.1 (3.53)	0.92	0.89-0.95	<0.001	<0.001
Hemoglobin concentration, g/dL	14.2 (1.24)	0.90	0.87-0.93	<0.001	<0.001

Data of blood biomarkers were z-score transformed (i.e., standardized data). False discovery rate (FDR) adjusted *P* values < 0.05 indicate statistically associations, as determined based on multiple-adjusted Cox proportional hazard models (blood biomarkers as independent variables, incident AMD as dependent variables). The HR corresponds to the risk folds of a new occurrence of AMD with per SD increase of blood biomarkers. Model was adjusted for baseline age and sex, race, BMI, smoking and alcohol drinking status, physical activity, education level, occupation status, stand height, history of hypertension, diabetes, stroke, angina, and heart attack.

Abbreviations: CI, confidence interval; HR, hazard ratio; LMR, lymphocyte-to-monocyte ratio; NLR, neutrophil-to-lymphocyte ratio; PLR, platelet-to-lymphocyte ratio; SII, systemic immune-inflammation index.

Table S11. Longitudinal associations between metabolites with the risk of incident AMD.

Metabolites (mean [SD])		Incident AMD			
		HR	95% CI	*P* value	*P* value FDR
Total Cholesterol, mmol/L	4.58 (0.93)	0.94	0.89-1.00	0.037	0.122
Total Cholesterol Minus HDL-C, mmol/L	3.27 (0.83)	0.94	0.89-0.99	0.019	0.120
Remnant Cholesterol, mmol/L	1.55 (0.41)	0.93	0.88-0.99	0.015	0.120
VLDL Cholesterol, mmol/L	0.72 (0.25)	0.94	0.89-0.99	0.017	0.120
Clinical LDL Cholesterol, mmol/L	2.52 (0.72)	0.94	0.89-1.00	0.036	0.121
LDL Cholesterol, mmol/L	1.72 (0.43)	0.94	0.89-0.99	0.030	0.120
HDL Cholesterol, mmol/L	1.31 (0.32)	1.01	0.95-1.07	0.823	0.926
Total Triglycerides, mmol/L	1.29 (0.57)	0.96	0.91-1.02	0.186	0.326
Triglycerides in VLDL, mmol/L	0.91 (0.48)	0.96	0.91-1.02	0.211	0.344
Triglycerides in LDL, mmol/L	0.14 (0.04)	0.96	0.91-1.02	0.177	0.312
Triglycerides in HDL, mmol/L	0.14 (0.05)	0.96	0.91-1.02	0.189	0.327
Total Phospholipids in Lipoprotein Particles, mmol/L	2.90 (0.47)	0.95	0.90-1.00	0.072	0.163
Phospholipids in VLDL, mmol/L	0.47 (0.18)	0.94	0.89-1.00	0.043	0.127
Phospholipids in LDL, mmol/L	0.60 (0.14)	0.95	0.90-1.00	0.044	0.127
Phospholipids in HDL, mmol/L	1.54 (0.32)	1.00	0.94-1.06	0.992	0.999
Total Esterified Cholesterol, mmol/L	3.32 (0.67)	0.94	0.89-1.00	0.048	0.127
Cholesteryl Esters in VLDL, mmol/L	0.43 (0.14)	0.93	0.88-0.99	0.014	0.120
Cholesteryl Esters in LDL, mmol/L	1.26 (0.32)	0.94	0.89-0.99	0.026	0.120
Cholesteryl Esters in HDL, mmol/L	1.02 (0.25)	1.01	0.95-1.07	0.722	0.870
Total Free Cholesterol, mmol/L	1.25 (0.27)	0.94	0.89-0.99	0.020	0.120
Free Cholesterol in VLDL, mmol/L	0.29 (0.11)	0.94	0.89-0.99	0.028	0.120
Free Cholesterol in LDL, mmol/L	0.46 (0.12)	0.95	0.9.0-1.00	0.053	0.137

Free Cholesterol in HDL, mmol/L	0.29 (0.07)	0.99	0.93-1.06	0.808	0.926
Total Lipids in Lipoprotein Particles, mmol/L	8.77 (1.63)	0.94	0.89-1.00	0.034	0.120
Total Lipids in VLDL, mmol/L	2.09 (0.85)	0.95	0.90-1.00	0.069	0.162
Total Lipids in LDL, mmol/L	2.46 (0.59)	0.94	0.89-0.99	0.032	0.120
Total Lipids in HDL, mmol/L	2.99 (0.64)	1.00	0.94-1.06	0.994	0.999
Total Concentration of Lipoprotein Particles, mmol/L	0.02 (<0.001)	0.98	0.93-1.04	0.541	0.713
Concentration of VLDL Particles, mmol/L	<0.001 (<0.001)	0.94	0.89-0.99	0.026	0.120
Concentration of LDL Particles, mmol/L	<0.001 (<0.001)	0.94	0.89-0.99	0.022	0.120
Concentration of HDL Particles, mmol/L	0.02 (<0.001)	0.99	0.94-1.05	0.819	0.926
Average Diameter for VLDL Particles, nm	38.6 (1.25)	0.96	0.91-1.02	0.217	0.345
Average Diameter for LDL Particles, nm	23.9 (0.09)	1.00	0.95-1.06	0.891	0.963
Average Diameter for HDL Particles, nm	9.65 (0.21)	1.01	0.95-1.08	0.705	0.856
Phosphoglycerides, mmol/L	2.26 (0.39)	0.96	0.90-1.01	0.122	0.245
Total Cholines, mmol/L	2.54 (0.41)	0.95	0.90-1.01	0.110	0.228
Phosphatidylcholines, mmol/L	2.08 (0.37)	0.95	0.90-1.01	0.086	0.192
Sphingomyelins, mmol/L	0.44 (0.07)	0.97	0.91-1.02	0.256	0.374
Apolipoprotein B, g/l	0.84 (0.20)	0.94	0.89-0.99	0.020	0.120
Apolipoprotein A1, g/l	1.44 (0.24)	1.00	0.94-1.06	0.924	0.964
Total Fatty Acids, mmol/L	11.9 (2.38)	0.96	0.90-1.01	0.107	0.224
Degree of Unsaturation, degree	1.36 (0.08)	0.99	0.94-1.05	0.858	0.955
Omega-3 Fatty Acids, mmol/L	0.53 (0.22)	0.97	0.92-1.02	0.206	0.344
Omega-6 Fatty Acids, mmol/L	4.46 (0.68)	0.95	0.90-1.00	0.054	0.137
Polyunsaturated Fatty Acids, mmol/L	4.99 (0.80)	0.94	0.89-1.00	0.044	0.127
Monounsaturated Fatty Acids, mmol/L	2.81 (0.81)	0.97	0.92-1.03	0.329	0.459
Saturated Fatty Acids, mmol/L	4.04 (0.94)	0.96	0.91-1.01	0.122	0.245

Linoleic Acid, mmol/L	3.42 (0.68)	0.95	0.90-1.00	0.047	0.127
Docosahexaenoic Acid, mmol/L	0.23 (0.08)	0.97	0.92-1.02	0.224	0.347
Alanine, mmol/L	0.30 (0.08)	0.98	0.93-1.03	0.485	0.643
Glutamine, mmol/L	0.53 (0.08)	1.01	0.95-1.06	0.827	0.926
Glycine, mmol/L	0.16 (0.06)	1.03	0.98-1.09	0.261	0.378
Histidine, mmol/L	0.06 (0.01)	1.00	0.95-1.06	0.919	0.964
Total Concentration of Branched-Chain Amino Acids (Leucine + Isoleucine + Valine), mmol/L	0.35 (0.08)	0.95	0.90-1.00	0.073	0.165
Isoleucine, mmol/L	0.05 (0.02)	0.97	0.92-1.03	0.307	0.431
Leucine, mmol/L	0.10 (0.03)	0.94	0.89-1.00	0.045	0.127
Valine, mmol/L	0.20 (0.04)	0.95	0.90-1.00	0.067	0.159
Phenylalanine, mmol/L	0.05 (0.01)	1.00	0.95-1.06	0.866	0.958
Tyrosine, mmol/L	0.06 (0.01)	0.98	0.93-1.04	0.545	0.713
Glucose, mmol/L	3.56 (1.11)	1.06	1.01-1.11	0.023	0.120
Lactate, mmol/L	3.80 (1.08)	1.00	0.95-1.06	0.929	0.964
Pyruvate, mmol/L	0.08 (0.03)	0.99	0.94-1.05	0.777	0.899
Citrate, mmol/L	0.06 (0.01)	0.99	0.94-1.04	0.683	0.846
3-Hydroxybutyrate, mmol/L	0.06 (0.06)	1.00	0.95-1.05	0.921	0.964
Acetate, mmol/L	0.02 (0.03)	1.02	0.97-1.06	0.421	0.574
Acetoacetate, mmol/L	0.01 (0.01)	1.01	0.96-1.06	0.619	0.782
Acetone, mmol/L	0.01 (0.01)	1.03	0.98-1.08	0.239	0.354
Creatinine, mmol/L	0.07 (0.01)	1.02	0.97-1.07	0.483	0.643
Albumin, g/l	39.0 (3.28)	0.93	0.88-0.98	0.005	0.099
Glycoprotein Acetyls, mmol/L	0.79 (0.11)	1.03	0.98-1.09	0.235	0.354
Concentration of Chylomicrons and Extremely Large VLDL Particles, mmol/L	<0.001 (<0.001)	0.97	0.92-1.03	0.285	0.410

Total Lipids in Chylomicrons and Extremely Large VLDL, mmol/L	0.23 (0.20)	0.97	0.92-1.03	0.297	0.424
Phospholipids in Chylomicrons and Extremely Large VLDL, mmol/L	0.04 (0.03)	0.97	0.92-1.03	0.306	0.431
Cholesterol in Chylomicrons and Extremely Large VLDL, mmol/L	0.06 (0.04)	0.97	0.91-1.02	0.219	0.345
Cholesteryl Esters in Chylomicrons and Extremely Large VLDL, mmol/L	0.03 (0.02)	0.96	0.91-1.02	0.210	0.344
Free Cholesterol in Chylomicrons and Extremely Large VLDL, mmol/L	0.03 (0.02)	0.97	0.91-1.02	0.267	0.354
Triglycerides in Chylomicrons and Extremely Large VLDL, mmol/L	0.14 (0.13)	0.97	0.92-1.03	0.331	0.459
Concentration of Very Large VLDL Particles, mmol/L	<0.001 (<0.001)	0.96	0.91-1.02	0.167	0.303
Total Lipids in Very Large VLDL, mmol/L	0.20 (0.13)	0.96	0.91-1.02	0.162	0.300
Phospholipids in Very Large VLDL, mmol/L	0.04 (0.03)	0.96	0.90-1.01	0.130	0.256
Cholesterol in Very Large VLDL, mmol/L	0.05 (0.03)	0.95	0.90-1.00	0.067	0.159
Cholesteryl Esters in Very Large VLDL, mmol/L	0.03 (0.01)	0.95	0.89-1.00	0.048	0.127
Free Cholesterol in Very Large VLDL, mmol/L	0.02 (0.01)	0.95	0.90-1.01	0.106	0.224
Triglycerides in Very Large VLDL, mmol/L	0.11 (0.08)	0.97	0.91-1.02	0.242	0.356
Concentration of Large VLDL Particles, mmol/L	<0.001 (<0.001)	0.96	0.91-1.01	0.140	0.266
Total Lipids in Large VLDL, mmol/L	0.33 (0.17)	0.96	0.91-1.01	0.137	0.263
Phospholipids in Large VLDL, mmol/L	0.07 (0.04)	0.96	0.91-1.01	0.132	0.256
Cholesterol in Large VLDL, mmol/L	0.10 (0.05)	0.95	0.90-1.00	0.071	0.163
Cholesteryl Esters in Large VLDL, mmol/L	0.05 (0.02)	0.95	0.90-1.00	0.046	0.127
Free Cholesterol in Large VLDL, mmol/L	0.05 (0.02)	0.96	0.90-1.01	0.114	0.235

Triglycerides in Large VLDL, mmol/L	0.16 (0.09)	0.96	0.91-1.02	0.207	0.344
Concentration of Medium VLDL Particles, mmol/L	<0.001 (<0.001)	0.94	0.89-0.99	0.029	0.120
Total Lipids in Medium VLDL, mmol/L	0.57 (0.20)	0.95	0.90-1.00	0.043	0.127
Phospholipids in Medium VLDL, mmol/L	0.13 (0.05)	0.94	0.89-0.99	0.024	0.120
Cholesterol in Medium VLDL, mmol/L	0.17 (0.07)	0.94	0.89-0.99	0.019	0.120
Cholesteryl Esters in Medium VLDL, mmol/L	0.09 (0.04)	0.94	0.89-0.99	0.026	0.120
Free Cholesterol in Medium VLDL, mmol/L	0.08 (0.03)	0.94	0.89-0.99	0.019	0.120
Triglycerides in Medium VLDL, mmol/L	0.27 (0.11)	0.96	0.91-1.02	0.175	0.312
Concentration of Small VLDL Particles, mmol/L	<0.001 (<0.001)	0.95	0.90-1.00	0.062	0.154
Total Lipids in Small VLDL, mmol/L	0.41 (0.13)	0.95	0.90-1.00	0.048	0.127
Phospholipids in Small VLDL, mmol/L	0.10 (0.03)	0.94	0.89-0.99	0.030	0.120
Cholesterol in Small VLDL, mmol/L	0.16 (0.05)	0.94	0.89-0.99	0.022	0.120
Cholesteryl Esters in Small VLDL, mmol/L	0.10 (0.03)	0.94	0.89-0.99	0.024	0.120
Free Cholesterol in Small VLDL, mmol/L	0.06 (0.02)	0.94	0.89-0.99	0.022	0.120
Triglycerides in Small VLDL, mmol/L	0.16 (0.06)	0.97	0.91-1.02	0.218	0.345
Concentration of Very Small VLDL Particles, mmol/L	<0.001 (<0.001)	0.93	0.88-0.98	0.010	0.120
Total Lipids in Very Small VLDL, mmol/L	0.36 (0.09)	0.93	0.88-0.98	0.011	0.120
Phospholipids in Very Small VLDL, mmol/L	0.10 (0.03)	0.94	0.89-0.99	0.017	0.120
Cholesterol in Very Small VLDL, mmol/L	0.19 (0.05)	0.93	0.88-0.98	0.006	0.105
Cholesteryl Esters in Very Small VLDL, mmol/L	0.13 (0.03)	0.93	0.88-0.98	0.007	0.105
Free Cholesterol in Very Small VLDL, mmol/L	0.06 (0.01)	0.93	0.88-0.98	0.008	0.107
Triglycerides in Very Small VLDL, mmol/L	0.07 (0.02)	0.96	0.91-1.02	0.161	0.300
Concentration of IDL Particles, mmol/L	<0.001 (<0.001)	0.94	0.89-1.00	0.033	0.120

Total Lipids in IDL, mmol/L	1.23 (0.29)	0.94	0.89-0.99	0.029	0.120
Phospholipids in IDL, mmol/L	0.29 (0.07)	0.93	0.88-0.99	0.015	0.120
Cholesterol in IDL, mmol/L	0.84 (0.21)	0.94	0.89-1.00	0.044	0.127
Cholesteryl Esters in IDL, mmol/L	0.62 (0.16)	0.95	0.89-1.00	0.054	0.137
Free Cholesterol in IDL, mmol/L	0.22 (0.06)	0.94	0.89-0.99	0.028	0.120
Triglycerides in IDL, mmol/L	0.10 (0.03)	0.96	0.91-1.01	0.130	0.256
Concentration of Large LDL Particles, mmol/L	<0.001 (<0.001)	0.94	0.89-0.99	0.029	0.120
Total Lipids in Large LDL, mmol/L	1.57 (0.37)	0.94	0.89-0.99	0.029	0.120
Phospholipids in Large LDL, mmol/L	0.35 (0.08)	0.94	0.89-1.00	0.034	0.120
Cholesterol in Large LDL, mmol/L	1.12 (0.28)	0.94	0.89-0.99	0.031	0.120
Cholesteryl Esters in Large LDL, mmol/L	0.82 (0.20)	0.94	0.89-0.99	0.027	0.120
Free Cholesterol in Large LDL, mmol/L	0.29 (0.08)	0.95	0.89-1.00	0.048	0.127
Triglycerides in Large LDL, mmol/L	0.10 (0.03)	0.96	0.91-1.02	0.164	0.300
Concentration of Medium LDL Particles, mmol/L	<0.001 (<0.001)	0.94	0.89-0.99	0.022	0.120
Total Lipids in Medium LDL, mmol/L	0.61 (0.16)	0.94	0.89-1.00	0.041	0.127
Phospholipids in Medium LDL, mmol/L	0.16 (0.04)	0.95	0.90-1.00	0.066	0.159
Cholesterol in Medium LDL, mmol/L	0.42 (0.12)	0.94	0.89-1.00	0.035	0.120
Cholesteryl Esters in Medium LDL, mmol/L	0.30 (0.09)	0.94	0.89-0.99	0.031	0.120
Free Cholesterol in Medium LDL, mmol/L	0.12 (0.03)	0.95	0.90-1.00	0.063	0.155
Triglycerides in Medium LDL, mmol/L	0.03 (0.01)	0.97	0.91-1.02	0.210	0.344
Concentration of Small LDL Particles, mmol/L	<0.001 (<0.001)	0.94	0.89-0.99	0.016	0.120
Total Lipids in Small LDL, mmol/L	0.28 (0.06)	0.95	0.90-1.00	0.047	0.127
Phospholipids in Small LDL, mmol/L	0.09 (0.02)	0.96	0.91-1.01	0.094	0.205
Cholesterol in Small LDL, mmol/L	0.18 (0.04)	0.94	0.89-1.00	0.039	0.127

Cholesteryl Esters in Small LDL, mmol/L	0.13 (0.03)	0.94	0.89-1.00	0.033	0.120
Free Cholesterol in Small LDL, mmol/L	0.05 (0.01)	0.95	0.91-1.01	0.093	0.204
Triglycerides in Small LDL, mmol/L	0.02 (0.01)	0.97	0.91-1.02	0.231	0.354
Concentration of Very Large HDL Particles, mmol/L	<0.001 (<0.001)	0.99	0.93-1.05	0.697	0.852
Total Lipids in Very Large HDL, mmol/L	0.17 (0.08)	1.00	0.94-1.06	0.906	0.964
Phospholipids in Very Large HDL, mmol/L	0.08 (0.04)	1.00	0.94-1.06	1.000	1.000
Cholesterol in Very Large HDL, mmol/L	0.08 (0.03)	1.00	0.94-1.06	0.887	0.963
Cholesteryl Esters in Very Large HDL, mmol/L	0.06 (0.03)	1.00	0.94-1.06	0.957	0.977
Free Cholesterol in Very Large HDL, mmol/L	0.02 (0.01)	0.99	0.93-1.04	0.648	0.813
Triglycerides in Very Large HDL, mmol/L	0.01 (<0.001)	0.96	0.91-1.01	0.141	0.266
Concentration of Large HDL Particles, mmol/L	<0.001 (<0.001)	1.00	0.94-1.07	0.885	0.963
Total Lipids in Large HDL, mmol/L	0.65 (0.32)	1.01	0.95-1.07	0.817	0.926
Phospholipids in Large HDL, mmol/L	0.32 (0.15)	1.01	0.95-1.07	0.774	0.899
Cholesterol in Large HDL, mmol/L	0.30 (0.17)	1.01	0.95-1.07	0.776	0.899
Cholesteryl Esters in Large HDL, mmol/L	0.23 (0.13)	1.01	0.95-1.07	0.731	0.87
Free Cholesterol in Large HDL, mmol/L	0.07 (0.04)	1.00	0.94-1.07	0.940	0.966
Triglycerides in Large HDL, mmol/L	0.03 (0.01)	0.97	0.91-1.02	0.222	0.346
Concentration of Medium HDL Particles, mmol/L	<0.001 (<0.001)	1.00	0.95-1.07	0.901	0.964
Total Lipids in Medium HDL, mmol/L	1.03 (0.22)	1.00	0.95-1.07	0.880	0.963
Phospholipids in Medium HDL, mmol/L	0.48 (0.10)	1.00	0.95-1.06	0.918	0.964
Cholesterol in Medium HDL, mmol/L	0.49 (0.12)	1.01	0.95-1.07	0.683	0.846
Cholesteryl Esters in Medium HDL, mmol/L	0.40 (0.10)	1.02	0.96-1.08	0.610	0.781
Free Cholesterol in Medium HDL, mmol/L	0.09 (0.02)	1.00	0.94-1.06	0.989	0.999
Triglycerides in Medium HDL, mmol/L	0.05 (0.02)	0.97	0.92-1.02	0.217	0.345

Concentration of Small HDL Particles, mmol/L	0.01 (<0.001)	0.99	0.93-1.04	0.599	0.772
Total Lipids in Small HDL, mmol/L	1.15 (0.16)	0.98	0.93-1.04	0.587	0.763
Phospholipids in Small HDL, mmol/L	0.66 (0.09)	0.99	0.93-1.04	0.618	0.782
Cholesterol in Small HDL, mmol/L	0.44 (0.06)	0.99	0.94-1.05	0.757	0.896
Cholesteryl Esters in Small HDL, mmol/L	0.33 (0.05)	1.00	0.95-1.05	0.934	0.964
Free Cholesterol in Small HDL, mmol/L	0.11 (0.02)	0.97	0.92-1.03	0.346	0.476
Triglycerides in Small HDL, mmol/L	0.05 (0.02)	0.97	0.91-1.02	0.239	0.354

Data of blood biomarkers were z-score transformed (i.e., standardized data). False discovery rate (FDR) adjusted *P* values < 0.05 indicate statistically associations, as determined based on multiple-adjusted Cox proportional hazard models (blood metabolites as independent variables, incident AMD as dependent variables). The HR corresponds to the risk folds of a new occurrence of AMD with per SD increase of blood biomarkers. Model was adjusted for baseline age and sex, race, BMI, smoking and alcohol drinking status, physical activity, education level, occupation status, stand height, history of hypertension, diabetes, stroke, angina, and heart attack.

Abbreviations: CI, confidence interval; HR, hazard ratio; HDL, high density lipoprotein; LDL, low density lipoprotein; VLDL, very low-density lipoprotein.

Table S12. The mediation effects of blood markers in the association between FVC and incident AMD risk.

**Inflammatory markers**	**Decrements in FVC**							
	**Direct effect**		**Indirect effect**		**Total effect**		**Prop**	***P* value**
	**Beta**	***P* value**	**Beta**	***P* value**	**Beta**	***P* value**		
Lymphocyte percentage, %	9.33E-04	0.004	2.15E-05	0.001	9.54E-04	0.003	2.249	0.026
C reactive protein, mg/L	8.44E-04	0.010	1.10E-04	<0.001	9.54E-04	0.003	11.54	0.015
SII, 10^9 cells/L	9.30E-04	0.004	2.43E-05	0.002	9.54E-04	0.003	2.543	0.032
**Erythrocyte-related markers**								
Red blood cell distribution width, %	8.99E-04	0.006	5.55E-05	<0.001	9.54E-04	0.003	5.812	0.015
Hematocrit percentage, %	9.65E-04	0.003	-1.06E-05	0.002	9.54E-04	0.003	-1.116	0.035
Haemoglobin concentration, g/dL	9.42E-04	0.004	1.23E-05	0.001	9.54E-04	0.003	1.292	0.029

Lung function data were minus-transformed. Prop was that the proportion of the total effect explained by the mediator. Model was adjusted for baseline age and sex, race, BMI, smoking and alcohol drinking status, physical activity, education level, occupation status, stand height, and history of hypertension, diabetes, stroke, angina, and heart attack. The direct, indirect, and total effects of lung function measures on outcome were indicated by the standardized beta estimates.

Abbreviations: FVC, forced vital capacity; SII, systemic immune-inflammation index; AMD, age-related macular degeneration.

Table S13. The mediation effects of blood markers in the association between FEV1 and incident AMD risk

**Inflammatory markers**	**Decrements in FEV1**							
	**Direct effect**		**Indirect effect**		**Total effect**		**Prop**	***P* value**
	**Beta**	***P* value**	**Beta**	***P* value**	**Beta**	***P* value**		
Neutrophil count, 10^9 cells/L	1.54E-03	<0.001	5.83E-05	0.021	1.59E-03	<0.001	3.657	0.035
Lymphocyte percentage, %	1.56E-03	<0.001	3.68E-05	0.001	1.59E-03	<0.001	2.308	0.005
SII, 10^9 cells/L	1.56E-03	<0.001	3.75E-05	0.002	1.59E-03	<0.001	2.355	0.008
C reactive protein, mg/L	1.48E-03	<0.001	1.15E-04	<0.001	1.59E-03	<0.001	7.213	<0.001
**Erythrocyte-related markers**								
Red blood cell distribution width, %	1.53E-03	<0.001	6.79E-05	<0.001	1.59E-03	<0.001	4.260	0.001
Hemoglobin concentration, g/dL	1.58E-03	<0.001	1.61E-05	<0.001	1.59E-03	<0.001	1.011	0.001

Lung function data were minus-transformed. Prop = the proportion of the total effect explained by the mediator. Model was adjusted for baseline age and sex, race, BMI, smoking and alcohol drinking status, physical activity, education level, occupation status, stand height, and history of hypertension, diabetes, stroke, angina, and heart attack. The direct, indirect, and total effects of lung function measures on outcome were indicated by the standardized beta estimates.

Abbreviations: FEV1, forced expiratory volume in 1 second; SII, systemic immune-inflammation index; AMD, age-related macular degeneration.

Table S14. The mediation effects of blood markers in the association between PEF and incident AMD risk

**Inflammatory markers**	**Decrements in PEF**							
	**Direct effect**		**Indirect effect**		**Total effect**		**Prop**	***P* value**
	**Beta**	***P* value**	**Beta**	***P* value**	**Beta**	***P* value**		
Neutrophil count, 10^9 cells/L	1.41E-03	<0.001	4.22E-05	0.017	1.45E-03	<0.001	2.911	0.030
Lymphocyte percentage, %	1.41E-03	<0.001	3.69E-05	0.001	1.45E-03	<0.001	2.547	0.004
C reactive protein, mg/L	1.37E-03	<0.001	7.89E-05	<0.001	1.45E-03	<0.001	5.450	0.001
SII, 10^9 cells/L	1.41E-03	<0.001	3.62E-05	0.002	1.45E-03	<0.001	2.500	0.008
**Erythrocyte-related markers**								
Erythrocyte count, 10^12 cells/L	1.43E-03	<0.001	1.54E-05	0.015	1.45E-03	<0.001	1.066	0.027
Red blood cell distribution width, %	1.40E-03	<0.001	4.62E-05	<0.001	1.45E-03	<0.001	3.188	0.001
Hematocrit percentage, %	1.42E-03	<0.001	2.58E-05	<0.001	1.45E-03	<0.001	1.778	<0.001
Hemoglobin concentration, g/dL	1.41E-03	<0.001	3.53E-05	<0.001	1.45E-03	<0.001	2.434	<0.001

Lung function data were minus-transformed. Prop was that the proportion of the total effect explained by the mediator. Model was adjusted for baseline age and sex, race, BMI, smoking and alcohol drinking status, physical activity, education level, occupation status, stand height, and history of hypertension, diabetes, stroke, angina, and heart attack. The direct, indirect, and total effects of lung function measures on outcome were indicated by the standardized beta estimates.

Abbreviations: PEF, peak expiratory flow; SII, systemic immune-inflammation index; AMD, age-related macular degeneration.

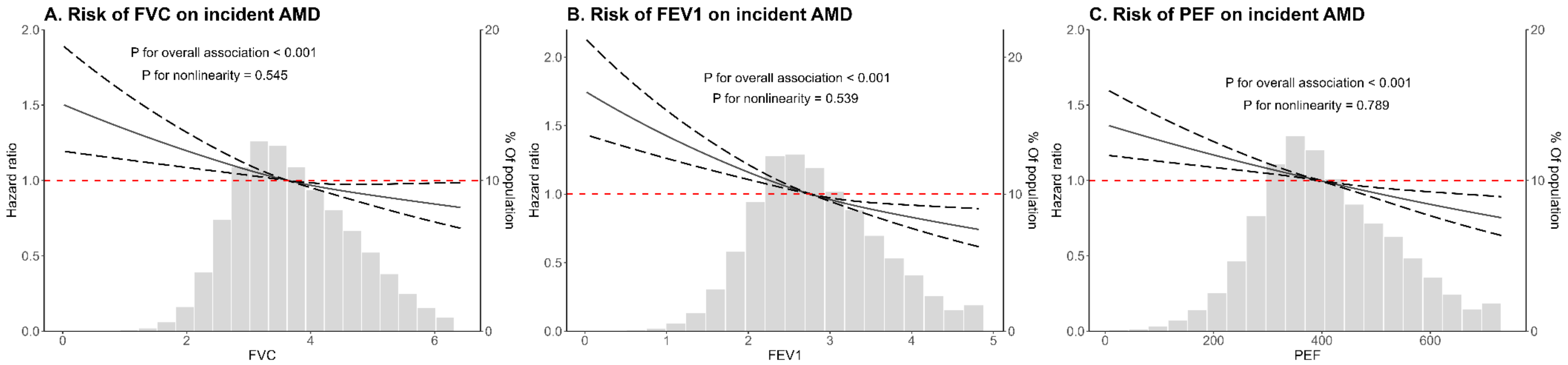

Figure S1. Association of lung function as a continuous scale with incident AMD. Restricted cubic splines consequences of the connection between incident AMD with FVC (A), FEV1 (B), and PEF (C).

HRs (solid line) and 95% CIs (dashed lines) from Cox proportional hazards regression models using restricted cubic splines. Covariates included baseline age, sex, race, smoking status, alcohol drinking, physical activity, BMI, education level, occupation status, stand height, and history of hypertension, diabetes, stroke, angina, and heart attack.

Abbreviations: FEV1, forced expiratory volume in 1 second; FVC, forced vital capacity; PEF, peak expiratory flow; AMD, Age-related macular degeneration.

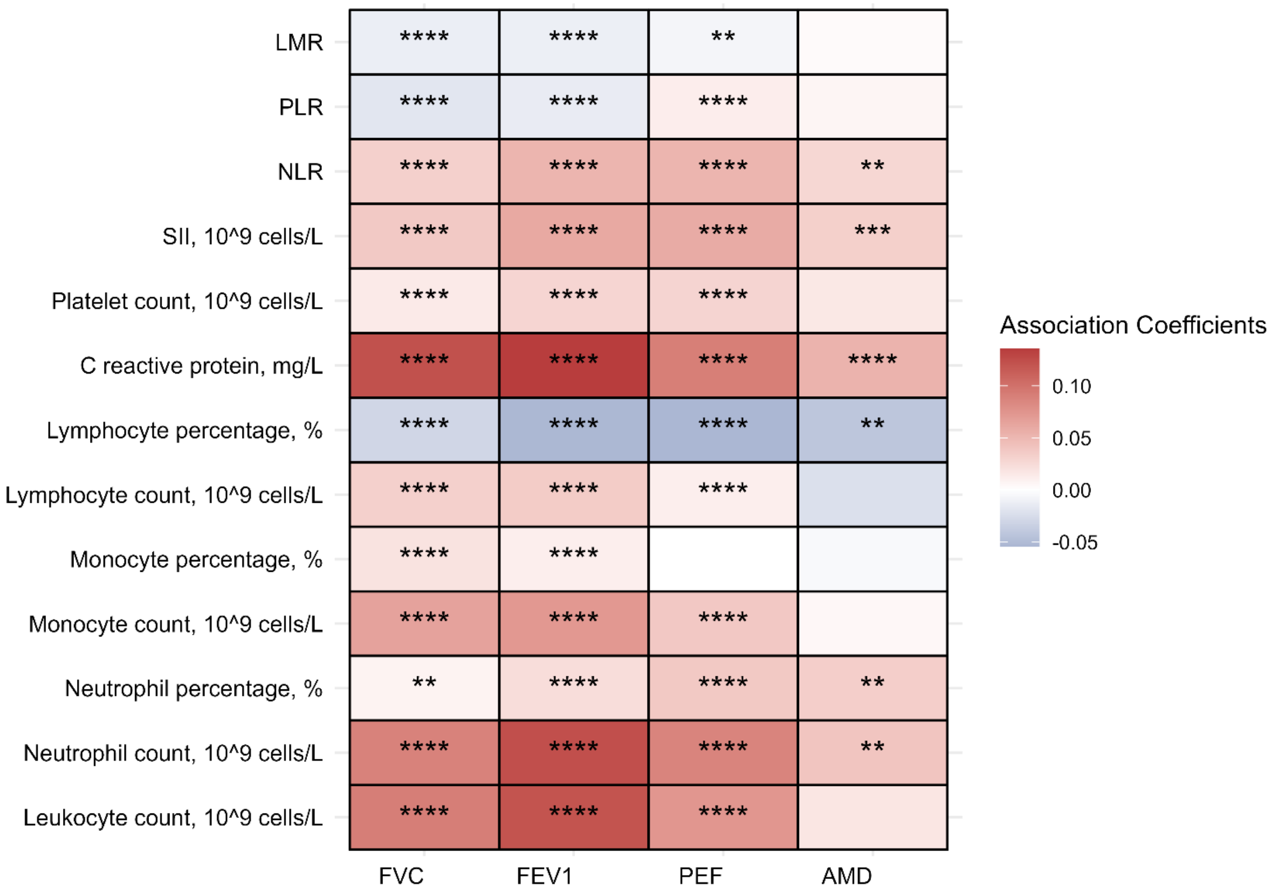

Figure S2. Heatmap of associations between blood inflammatory markers with lung function and incident AMD.

Lung function data were minus-transformed. False discovery rate (FDR) adjusted *P* values <0.05 indicate statistically associations, as determined based on multiple-adjusted linear models (lung function as independent variables, inflammatory markers as dependent variables) or Cox proportional hazard models (inflammatory markers as independent variables, incident AMD as dependent variables). ****P values <0.0001, ***P values <0.001, **P values <0.01, *P values <0.05.

Abbreviations: FEV1, forced expiratory volume in 1 second; FVC, forced vital capacity; LMR, lymphocyte-to-monocyte ratio; NLR, neutrophil-to-lymphocyte ratio; PEF, peak expiratory flow; PLR, platelet-to-lymphocyte ratio; SII, systemic immune-inflammation index; AMD, age-related macular degeneration.

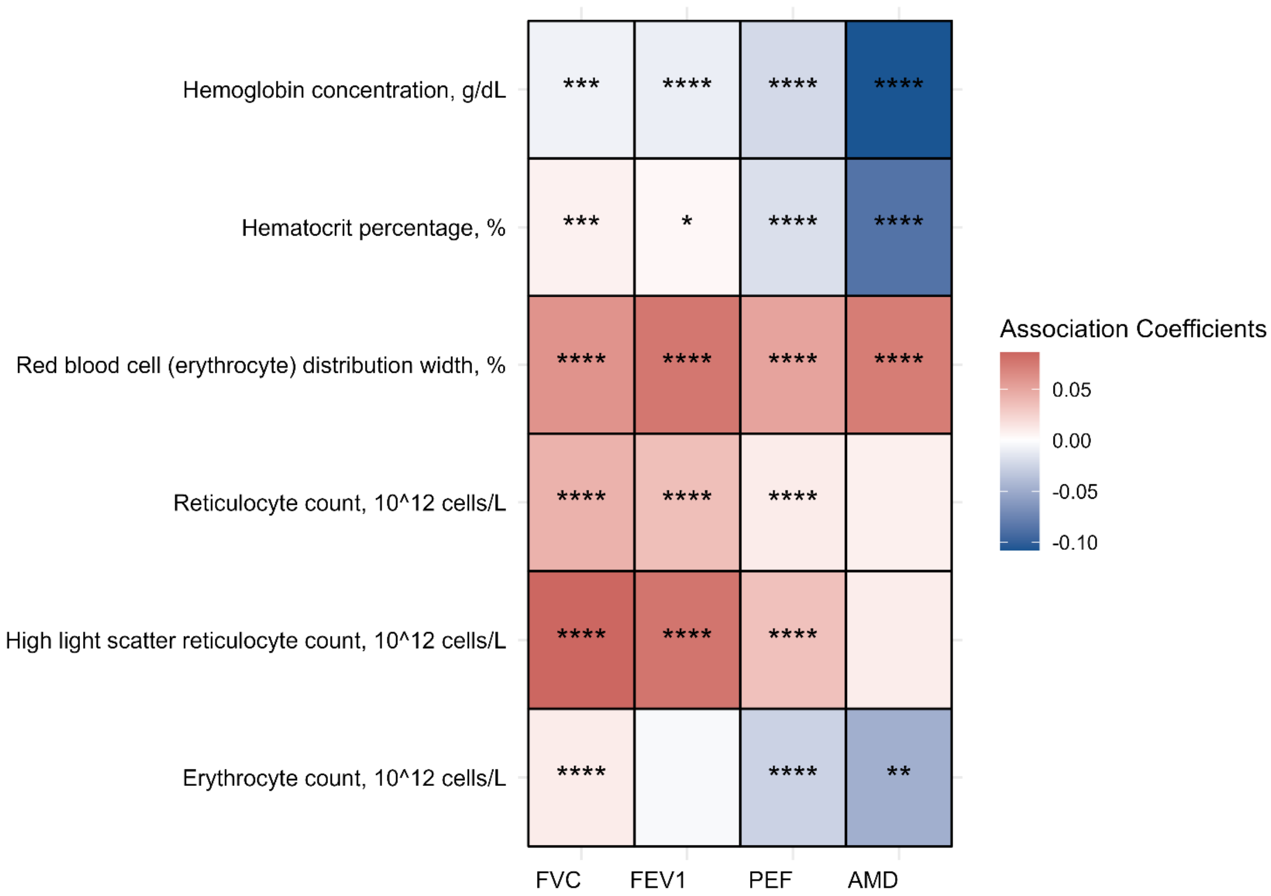

Figure S3. Heatmap of associations between erythrocyte-related measures with lung function and incident AMD.

Lung function data were minus-transformed. False discovery rate (FDR) adjusted *P* values <0.05 indicate statistically associations, as determined based on multiple-adjusted linear models (lung function as independent variables, erythrocyte-related measures as dependent variables) or Cox proportional hazard models (erythrocyte-related measures as independent variables, incident AMD as dependent variables). ****P values <0.0001, ***P values <0.001, **P values <0.01, *P values <0.05.

Abbreviations: FEV1, forced expiratory volume in 1 second; FVC, forced vital capacity; PEF, peak expiratory flow; AMD, age-related macular degeneration.

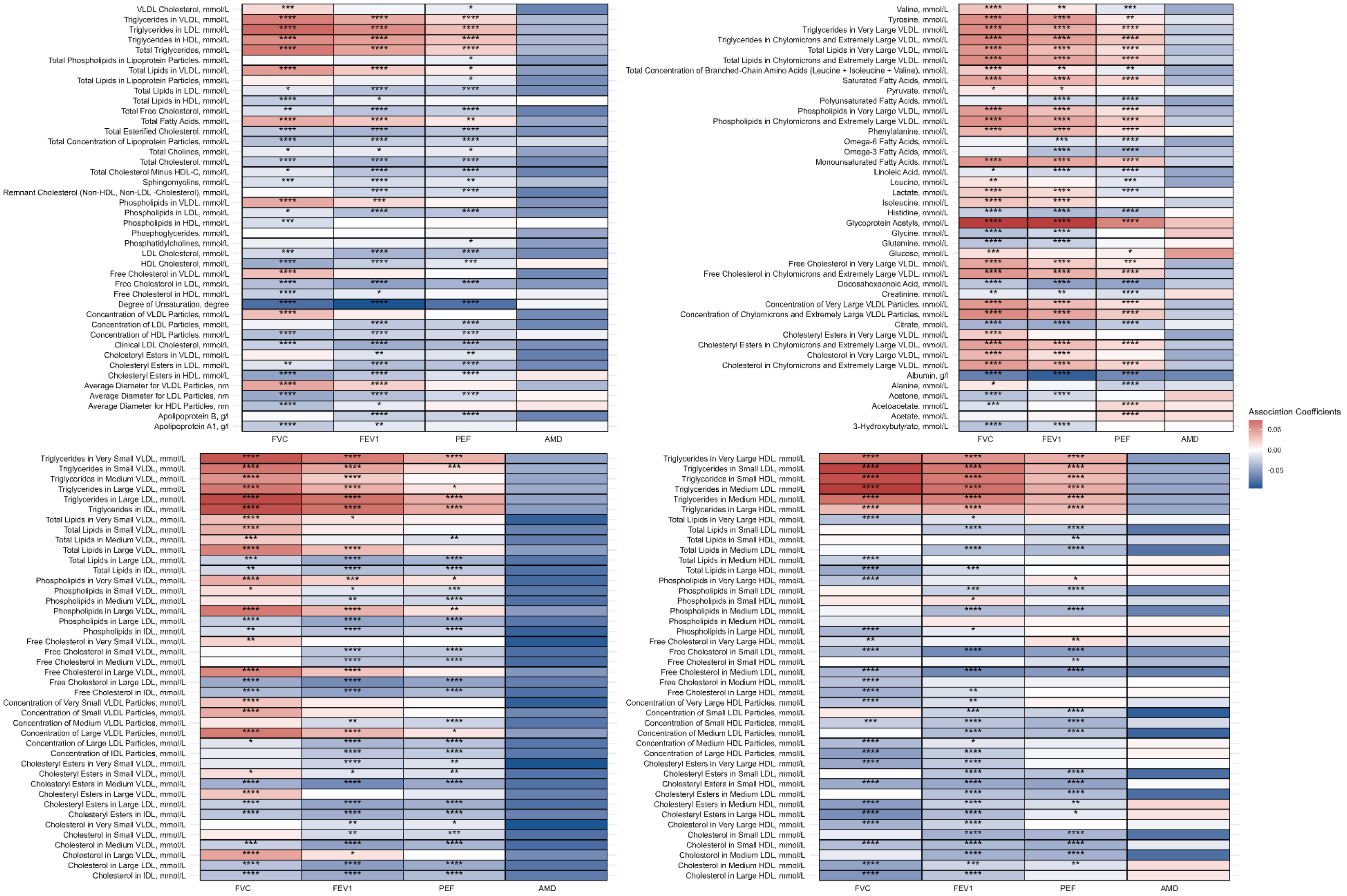

Figure S4. Heatmap of associations between blood metabolites with lung function and incident AMD.

Lung function data were minus-transformed. False discovery rate (FDR) adjusted *P* values <0.05 indicate statistically associations, as determined based on multiple-adjusted linear models (lung function as independent variables, blood metabolites as dependent variables) or Cox proportional hazard models (blood metabolites as independent variables, incident AMD as dependent variables). ****P values <0.0001, ***P values <0.001, **P values <0.01, *P values <0.05.

Abbreviations: FEV1, forced expiratory volume in 1 second; FVC, forced vital capacity; HDL, high density lipoprotein; LDL, low density lipoprotein; PEF, peak expiratory flow; VLDL, very low-density lipoprotein; AMD, age-related macular degeneration.
